# Boosting the Strength and Toughness of Polymer Blends via Ligand‐Modulated MOFs

**DOI:** 10.1002/advs.202407593

**Published:** 2024-10-16

**Authors:** Tairong Kuang, Hongxin Guo, Wei Guo, Wenxian Liu, Wei Li, Mohammad Reza Saeb, Mohammad Vatankhah‐Varnosfaderani, Sergei S. Sheiko

**Affiliations:** ^1^ Functional Polymers & Advanced Materials (FPAM) Lab Zhejiang Key Laboratory of Plastic Modification and Processing Technology College of Materials Science and Engineering Zhejiang University of Technology Hangzhou Zhejiang 310014 P. R. China; ^2^ Institute for Chemical Reaction Design and Discovery (WPI‐ICReDD) Hokkaido University Sapporo 001–0021 Japan; ^3^ Suzhou Laboratory Suzhou Jiangsu 215123 P. R. China; ^4^ Department of Pharmaceutical Chemistry Medical University of Gdańsk J. Hallera 107 Gdańsk 80–416 Poland; ^5^ Department of Chemistry University of North Carolina at Chapel Hill Chapel Hill NC 27599 USA

**Keywords:** amphiphilic nanoparticle, metal–organic framework, molecular dynamics simulations, polymer blends, strength and toughness

## Abstract

Mechanically robust and tough polymeric materials are in high demand for applications ranging from flexible electronics to aerospace. However, achieving both high toughness and strength in polymers remains a significant challenge due to their inherently contradictory nature. Here, a universal strategy for enhancing the toughness and strength of polymer blends using ligand‐modulated metal–organic framework (MOF) nanoparticles is presented, which are engineered to have adjustable hydrophilicity and lipophilicity by varying the types and ratios of ligands. Molecular dynamics (MD) simulations demonstrate that these nanoparticles can effectively regulate the interfaces between chemically distinct polymers based on their amphiphilicity. Remarkably, a mere 0.1 wt.% of MOF nanoparticles with optimized amphiphilicity (ML‐MOF_(5:5)_) delivered ≈1.1‐ and ≈34.1‐fold increase in strength and toughness of poly (lactic acid) (PLA)/poly (butylene succinate) (PBS) blend, respectively. Moreover, these amphiphilicity‐tailorable MOF nanoparticles universally enhance the mechanical properties of various polymer blends, such as polypropylene (PP)/polyethylene (PE), PP/polystyrene (PS), PLA/poly (butylene adipate‐co‐terephthalate) (PBAT), and PLA/polycaprolactone (PCL)/PBS. This simple universal method offers significant potential for strengthening and toughening various polymer blends.

## Graphical abstract

A universal strategy is reported to boost the toughness and strength of polymer blends using ligand‐modulated metal–organic framework (MOF) nanoparticles with tunable hydrophilicity and lipophilicity. Molecular dynamics simulations reveal the volcano‐like dependency between the amphiphilicity of compatibilizers and compatibilizing effectiveness.

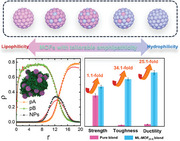

## 1 Introduction

Polymeric materials possessing both high strength and toughness are crucial, attracting significant interest in varied fields such as aerospace, smart architecture, bioengineering, and flexible electronics.^[^ ^1^, ^2^, ^3^, ^4^, ^5^, ^6^ ^]^ However, a fundamental challenge lies in the inherent contradiction between strength (the ability to bear mechanical loads) and toughness (the capacity to resist fracture).^[^ ^7^, ^8^, ^9^ ^]^ Strength in polymeric materials is primarily determined by molecular arrangement and bond strength, which are essential for materials with low molecular weight. In contrast, toughness relates to energy dissipation via complex mechanisms, extending beyond simple defect resistance.^[^ ^10^ ^]^ High strength, derived from strong chemical bonds and orderly structures, does not inherently result in toughness, which also depends on a material's modulus and deformability, supported by energy dissipation processes such as dynamic or sacrificial bonding.^[^ ^10^ ^]^ This inherent disparity underscores the challenge of engineering polymers to simultaneously exhibit high strength and toughness, requiring universal solutions to reconcile these conflicting properties.^[^ ^11^, ^12^, ^13^ ^]^

Advances in polymer science have led to the development of various strategies for enhancing the strength and toughness of polymeric materials, such as cross‐linking network design^[^ ^14^, ^15^ ^]^ and copolymerization.^[^ ^16^, ^17^ ^]^ Cross‐linking network design enhances the strength and toughness of polymeric materials by linking polymer chains into a cohesive 3D matrix. This cross‐linking process reduces chain mobility, which in turn increases the modulus and thermal stability of the material.^[^ ^18^ ^]^ Traditional covalent cross‐linking, although effective in strengthening polymers, often reduces their extensibility due to the rigid and permanent nature of covalent bonds.^[^ ^19^, ^20^ ^]^ Noncovalent cross‐linking, utilizing reversible interactions such as hydrogen bonding, ionic bonding, metal coordination, van der Waals forces, and π‐π interactions, allows materials to dynamically absorb and dissipate energy, maintaining integrity under mechanical stresses.^[^ ^21^ ^]^ Copolymerization introduces structural diversity and tailored microphase separation, enhancing material properties by integrating varied monomeric units into a single polymer chain.^[^ ^22^, ^23^ ^]^ Despite their success, these processes present challenges such as complex processing, increased production costs, trade‐offs between properties like strength and ductility, recyclability issues, and scalability concerns.

Polymer blends offer a compelling alternative due to simpler processing, adjustable properties through composition modifications, and better recyclability, providing a sustainable solution to the limitations of single polymer modifications.^[^ ^24^, ^25^, ^26^, ^27^, ^28^, ^29^ ^]^ Unfortunately, achieving homogeneous blends is challenging due to the immiscibility of many polymer pairs, leading to phase separation and compromised material performance. The development of compatibilizers with amphiphilic characteristics, such as carbon nanotubes (CNTs)^[^ ^29^ ^]^ and Janus particles,^[^ ^30^, ^31^ ^]^ presents new opportunities for improving polymer blend compatibilization. These compatibilizers consist of two components, one of which is attracted to one polymer, while the other interacts with the other polymer.^[^ ^25^, ^32^ ^]^ They can reduce the interfacial energy between chemically different polymers, facilitating blending and enhancing the thermodynamic stability of polymer blends.^[^ ^31^, ^33^, ^34^ ^]^ The bridging effect of compatibilizers depends on their amphiphilicity, which reduces interfacial tension and improves the dispersion of polymer phases.^[^ ^35^, ^36^ ^]^ Despite these advances, precise regulation of the hydrophilic and/or oleophilic components of amphiphilic compatibilizers and their impact on interfacial structure and compatibilization behavior remain underexplored.

**Figure 1:**
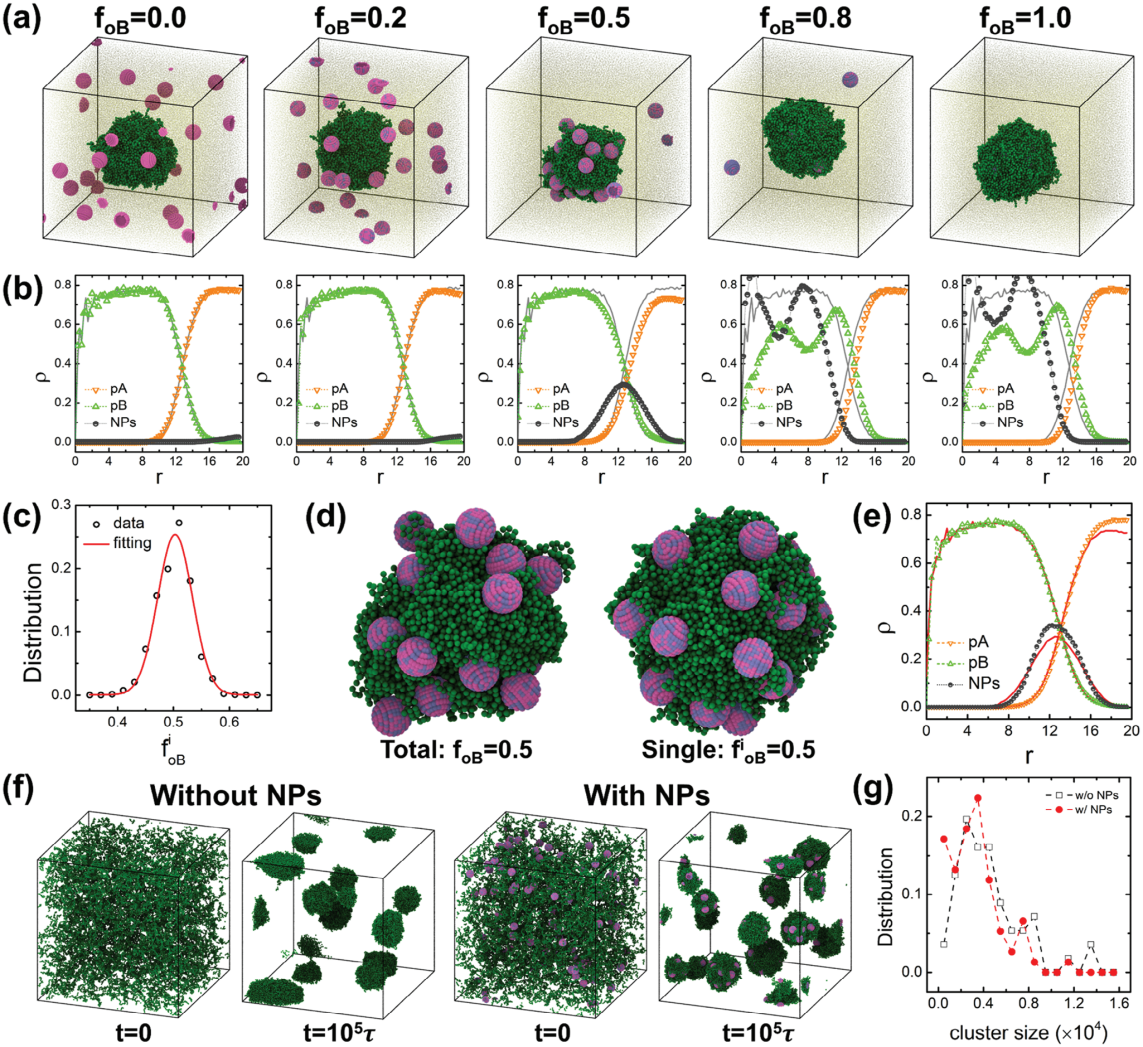
MD simulation study of the phase segregation behaviors in the mixture system consisting of a binary immiscible blend of homopolymers and amphiphilic particles. a) Snapshots of mixture systems containing amphiphilic particles (with a total number of *n*_NP_ =  20). The overall fraction of oB organic ligands, $f_{\mathrm{oB}}=\sum_{i}n_{\mathrm{oB}}^{i}\;/\left(n_{o}n_{\mathrm{NP}}\right)$, $i\;=\;1,\text{…},n_{\mathrm{NP}}$, is controlled. b) Corresponding radial density profiles of the polymer and amphiphilic particle beads, with the origin located at the center of mass of the B domain, for systems in (a). The density profiles of the corresponding neat polymer blend (Figure S1, Supporting Information) are also shown in gray lines for comparison. (c) Distribution of the oB fraction of individual amphiphilic particles, $f_{\mathrm{oB}}^{i}=n_{\mathrm{oB}}^{i}\;/n_{o}$, in the case of *f*_oB_ =  0.5, which exhibits a Gaussian distribution. The fitted standard deviation is 0.032, close to the predicted value of [*f*_oB_(1 − *f*_oB_)/*n*_o_]^1/2^ ≈ 0.033. d) Typical snapshots of B domains surrounded by amphiphilic particles in systems of *f*_oB_ =  0.5 and $f_{\mathrm{oB}}^{i}=0.5$, respectively, where in the latter case the oB fraction is fixed for individual amphiphilic particles. e) Comparison between the radial density profiles of $f_{\mathrm{oB}}^{i}=0.5$ (symbols) with *f*_oB_ =  0.5 (solid red lines), indicating that the density profile of amphiphilic particle beads is more concentrated at $f_{\mathrm{oB}}^{i}=0.5$. f) Snapshots of initial and phase‐separated configurations for systems without and with amphiphilic particles (*n*_NP_ =  100 and  *f*_oB_ =  0.5). g) Cluster size distribution of polymer domains in the two systems in (f).

In this work, we employed coarse‐grained molecular dynamics (MD) simulations to investigate the effect of compatibilizer amphiphilicity on the interface of incompatible components (**Figure** **1**). Our results indicate that the spatial distribution of compatibilizer within the system correlates with their amphiphilic behavior. Specifically, particles with optimized amphiphilicity effectively distribute at the interface of incompatible polymers, resulting in superior compatibilizing properties. Traditional compatibilization particles face challenges in accurately balancing hydrophilic and oleophilic components. In contrast, metal–organic frameworks (MOFs) offer a sophisticated means to fine‐tune this balance through precise regulation of mixed organic ligands.^[^ ^37^, ^38^, ^39^, ^40^, ^41^, ^42^ ^]^ Leveraging this capability, we designed ligand‐modulated MOF nanoparticles as compatibilizers, where the ratio of oleophilic ligand (BDC, 1, 4‐benzenedicarboxylate) to hydrophilic ligand (NH_2_‐BDC, 2‐amino‐1,4‐benzenedicarboxylate) can be modulated to enhance the interfaces of incompatible polymer blends. Specifically, the NiCo‐BDC/NH_2_‐BDC MOF was chosen as a proof of concept, due to its unique lamellar structure, high stability, amphiphilicity tunability, and cost efficiency. The optimized mixed‐ligand MOF (ML‐MOF_(5:5)_) nanoparticle exhibits impressive compatibilizing properties, delivering ≈1.1‐fold strength enhancement and ≈34.1‐fold toughness enhancement for the poly (lactic acid)/poly (butylene succinate) (PLA/PBS) blend at a low additive mass fraction of 0.1%. Furthermore, ML‐MOF_(5:5)_ can act as a universal compatibilizer for preparing various high‐strength, high‐toughness polymer blends, including PLA/poly (butylene adipate‐*co*‐terephthalate) (PBAT), PLA/PBS/poly‐*ε*‐caprolactone (PCL), polypropylene (PP)/polyethylene (PE) and PP/polystyrene (PS). This work presents a novel and universal approach to modulating incompatible interfaces for creating polymer blend materials with high strength and toughness.

## 2 Results and Discussion

### 2.1 MD Simulations of the Impact of Amphiphilic Particles on Incompatible Systems

The system setup illustrated in Figure S1 and Table S1 (Supporting Information) shows A and B homopolymers and a phase‐separated blend where B polymer (pB) form an isolated domain inside an A polymer (pA) matrix, with amphiphilic nanoparticles composed of organic affinity units oA and oB at varying ratios. Figure 1a presents typical simulation snapshots for mixtures of immiscible pA and pB polymer blends with nanoparticles (NPs) that have randomly distributed oA and oB affinity units. The total oB fraction, *f*_oB_, varies from 0 to 1.

Figure 1b shows the associated radial density profiles, with the origin located at the center of mass of the segregated pB phase domain, to characterize the distribution of NPs. Results indicate that amphiphilic NPs of *f*_oB_ =  0.5 act as surfactants, residing preferentially at the interface between pA and pB domains. In contrast, NPs are mostly distributed inside the pA domain for *f*_oB_ < 0.5 and inside the pB domain for *f*_oB_ > 0.5. These behaviors are attributed to the enthalpic considerations, where the total attraction overcomes the total repulsion exerted by neighboring polymer beads on the amphiphilic NPs that their staying in the preferred polymer domain (determined by the majority affinity units) leads to lower free energy.

For the random assignment of oA and oB affinity units in amphiphilic particles according to *f*_oB_, the distribution of the oB fraction in individual NP, $f_{\mathrm{oB}}^{i}$, follows a Gaussian distribution with a standard deviation σ_sd_ = [*f*_oB_(1 − *f*_oB_)/*n*_o_]^1/2^, where *n*_o_ = *n*_oA_  + *n*_oB_ is the total number of affinity units in a single amphiphilic particle (Figure 1c). Reducing σ_sd_ enhances the localization of NPs at the interface, as demonstrated by comparing systems with *f*_oB_ =  0.5 and a control group with $f_{\mathrm{oB}}^{i}=0.5$ (i.e., σ_sd_ =  0). Figure 1d presents typical snapshots of pB phase domains surrounded by NPs in these systems, indicating a more regulated pB domain surface at $f_{\mathrm{oB}}^{i}=0.5$. This is further captured by the radial density profiles in Figure 1e, showing a more concentrated NP density around the pA and pB interface, with sharper polymer density profiles at the interface.

To explore the potential of using simply assembled amphiphilic NPs as compatibilizers, we examined their effects on the phase segregation behavior and mechanical properties of the immiscible polymer blends, focusing on *f*_oB_ =  0.5 for its generality. Figure 1f compares phase separation in melt systems with and without amphiphilic NPs, starting from random homogeneous configurations. The presence of NPs at the interface imparts a pinning effect, stabilizing the phase domain and slowing domain coalescence, thereby reducing domain size, as shown by the cluster size distribution shift toward smaller values in Figure 1g. The enlarged dispersion and interfacial area are desired for enhancing the mechanical performance. Overall, the MD simulation results demonstrate that the distribution of amphiphilic NPs in an immiscible polymer blend can be controlled by tuning particle amphiphilicity. NPs with an amphiphilic ratio of 5:5 exhibit a good compatibilization effect, promising reinforcement for polymer blends.

### 2.2 Compatibilization Effect of ML‐MOF Nanoparticles in Immiscible PLA/PBS Blend

**Figure 2:**
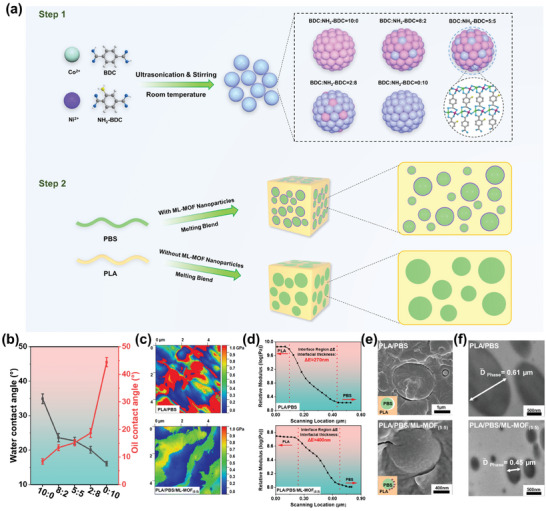
Preparation and morphological characterization of polymer blends with mixed‐ligand metal–organic framework (ML‐MOF) nanoparticles: a) Schematic illustration of the synthesis of ML‐MOF nanoparticle compatibilizers and the fabrication of PLA/PBS blends with and without ML‐MOF nanoparticles. b) Water and oil contact angles of ML‐MOFs with varying BDC/NH_2_‐BDC ligand ratios. c) Distribution of Young's modulus in PLA/PBS and PLA/PBS/ML‐MOF_(5:5)_ blends. d) Variation of Young's modulus as a function of scanning location across different polymer phases in PLA/PBS and PLA/PBS/ML‐MOF_(5:5)_ blends. e) SEM images of PLA/PBS and PLA/PBS/ML‐MOF_(5:5)_ blends. f) TEM images of PLA/PBS and PLA/PBS/ML‐MOF_(5:5)_ blends.

Drawing insights from MD simulations, we synthesized amphiphilic nanoparticles using metal–organic frameworks (MOFs) due to their high tunability and structural adaptability. The amphiphilicity of these nanoparticles can be precisely controlled by adjusting the ratios of metal ions and surface‐modified ligands.^[^ ^43^, ^44^, ^45^ ^]^ Specifically, we synthesized mixed‐ligand MOF (ML‐MOF) nanoparticles with varying ratios of oleophilic ligand (1, 4‐benzenedicarboxylate, BDC) to hydrophilic ligands (2‐amino‐1,4‐benzenedicarboxylate, NH_2_‐BDC). The ligands BDC and/or NH_2_‐BDC were reacted with Co^2+^ and Ni^2+^ under ultrasonic conditions at room temperature, yielding NiCo‐BDC/NH_2_BDC nanoparticles (**Figure** **2**a). The synthesized ML‐MOF nanoparticles were categorized based on their BDC/NH_2_‐BDC ratios: ML‐MOF_(10:0)_, ML‐MOF_(8:2)_, ML‐MOF_(5:5)_, ML‐MOF_(2:8)_, and ML‐MOF_(0:10)_.

Several characterization techniques were employed to analyze the synthesized ML‐MOF nanoparticles, confirming their successful synthesis and intended design. X‐ray diffraction analysis (XRD) analysis revealed a peak at 8.9°, indicative of the (200) facet and confirming the synthesis of Ni‐Co‐based bimetallic MOFs (Figure S2, Supporting Information). Fourier‐transform infrared spectroscopy (FTIR) results showed characteristic absorption peaks at 1049 and 1240 cm^−1^ associated with the C─N bond, and a notable peak at 3335 cm^−1^ corresponding to stretching vibration of the NH_2_ group (Figure S3, Supporting Information). Moreover, Raman spectroscopy and X‐ray photoelectron spectroscopy (XPS) results (Figures S4–S9, Supporting Information) provide additional confirmation of the successful synthesis of ML‐MOFs. Zeta potential analysis indicated a gradual increase in potential with a rising NH_2_‐BDC ratio, suggesting a proportional increase in NH_2_ groups and confirming the synthesis of MOFs with varied amphiphilic ratios (Figure S10, Supporting Information). Scanning electron microscopy (SEM) images displayed the granular morphology of the ML‐MOF nanoparticles, and transmission electron microscopy (TEM) provided further insights into their microstructure (Figure S11, Supporting Information). Elemental mapping demonstrated the uniform distribution of Ni, Co, N, C, and O within the nanoparticles, and the elemental composition showed an ≈1:1 ratio between Ni and Co, consistent with the initial synthesis conditions. These comprehensive results robustly confirm the successful synthesis and design of the ML‐MOF nanoparticles.

The synthesized ML‐MOF nanoparticles were incorporated into the immiscible PLA/PBS blend with a weight ratio of 70:30 at a concentration of a mere 0.1 wt.% to evaluate their impact on polymer compatibility and overall performance (Figure 2a). FTIR analysis confirmed the incorporation of NPs, revealing distinct absorption peaks for the C─N bond at 1245.8 and the C─H bond in the aromatic ring at 1150.8 cm^−1^ (Figure S12, Supporting Information).^[^ ^46^, ^47^ ^]^ The amphiphilic properties of the synthesized MOFs were characterized by measuring the contact angles with water and organic solvents^[^ ^48^ ^]^ such as n‐hexane^[^ ^49^ ^]^. The wettability of the ML‐MOF nanoparticles varied with the ratio of hydrophilic to oleophilic ligands, as indicated by changes in water and oil contact angles (Figure 2b; Figure S13, Supporting Information). An increase in oleophilic ligands led to a decrease in the oil contact angle to 8.4°, while an increase in hydrophilic ligands resulted in a decrease in the water contact angle to 16.1°. Notably, the ML‐MOF_(5:5)_ nanoparticle exhibited balanced hydrophilicity and oleophilicity. SEM images of PLA/PBS and PLA/PBS/ML‐MOF_(5:5)_ blends demonstrated significant morphological differences (Figure 2e; Figure S14, Supporting Information). The addition of ML‐MOF_(5:5)_ nanoparticle caused a marked reduction in the PBS domain size, with the average diameter decreasing from 2.1 to 0.5 µm, as measured from the images (Figure S15, Supporting Information). This reduction in domain size was further supported by TEM images (Figure 2f), indicating that ML‐MOF_(5:5)_ nanoparticle enhances PBS dispersion within PLA and stabilizes the domain structure, consistent with the simulation findings in Figure 1f.

The PeakForce Quantitative Nanomechanics (QNM) mode of atomic force microscopy (AFM) was utilized to determine the modulus distribution in the blends, with color variations from blue to red indicating low to high modulus values, respectively (Figure 2c). The blue regions represent the PBS component with a lower modulus, while the green and red regions correspond to the PLA component with a higher modulus.^[^ ^50^ ^]^ Notably, the introduction of 0.1 wt.% ML‐MOF_(5:5)_ nanoparticle diminished the modulus disparity between PLA and PBS phases, resulting in a more homogeneous modulus distribution across the polymer phases, thereby reducing stress concentration and hindering blend fractures.^[^ ^51^ ^]^ Additionally, the inclusion of ML‐MOF_(5:5)_ nanoparticles broadened the interfacial width between PLA and PBS from ≈270 to ≈400 nm (Figure 2d), aligning well with the simulation result in Figure 1b, and indicating effective interface smearing and compatibilization behavior within the blend system.^[^ ^52^ ^]^ Furthermore, dynamic thermomechanical analysis (DMA) focused on the glass transition temperature (*T_g_*) revealed a *T_g_* of ≈79.5 °C for PLA/PBS/ML‐MOF_(5:5)_ blend, lower than *T_g_* of ≈84.6 °C of the PLA/PBS blend (Figure S16, Supporting Information), confirming the enhanced interfacial compatibility induced by the ML‐MOF_(5:5)_ nanoparticle.

### 2.3 Mechanical Enhancement by ML‐MOF Nanoparticles in Immiscible PLA/PBS Blend

**Figure 3:**
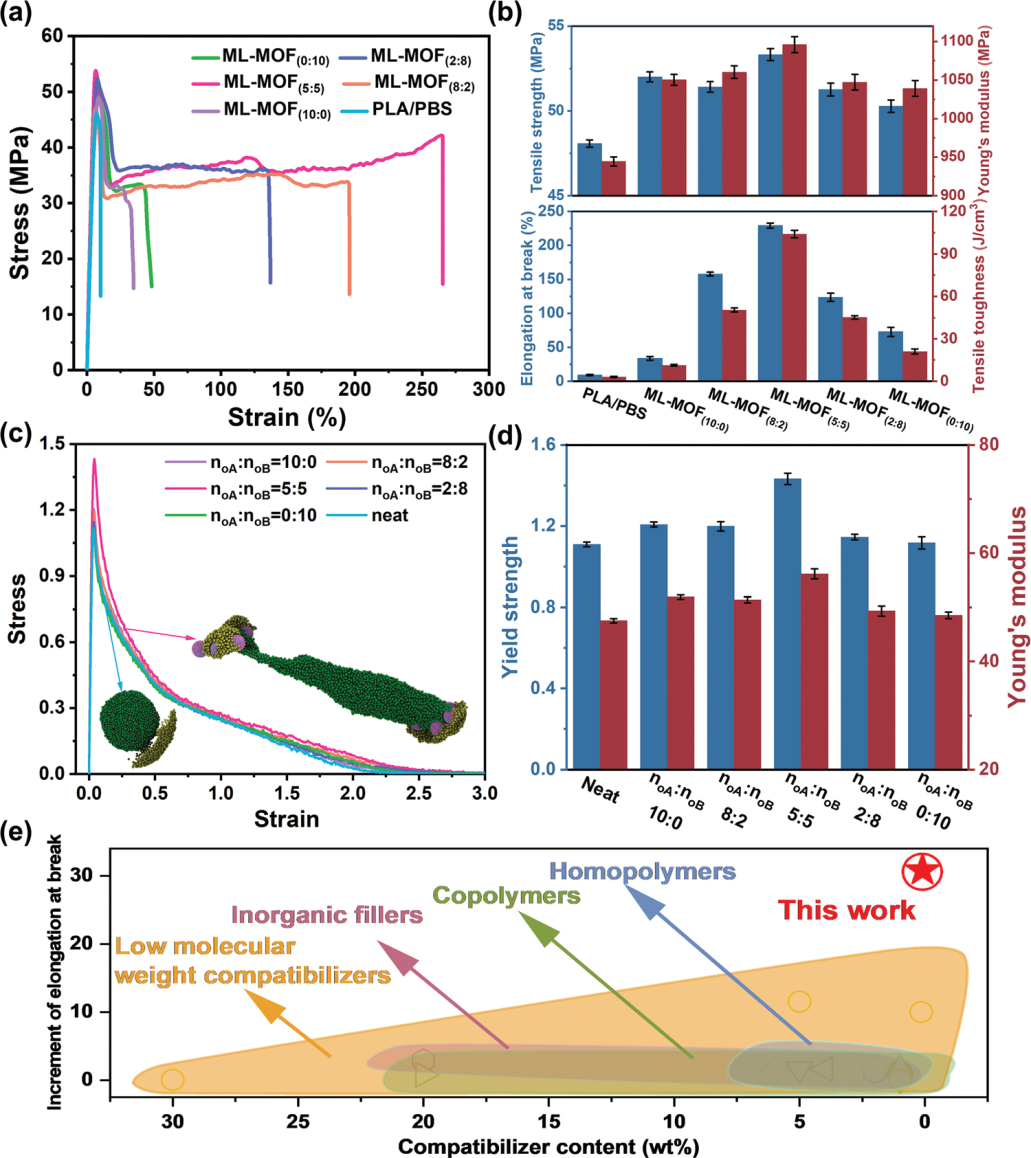
Experimental and simulated mechanical properties of various samples: a) Stress‐strain curves from tensile tests for PLA/PBS blends with different ML‐MOFs (0.1 wt.%). b) Mechanical properties derived from the stress‐strain curves in (a). c) Stress‐strain curves of corresponding model systems from uniaxial deformation simulations. The inset shows typical configurations of the pB domain at strains indicated by the arrows, highlighting the pinning effect of amphiphilic nanoparticles that stretches the polymer phase domain under extension. d) Yield strength and Young's modulus obtained from simulated curves in (c). e) Comparison of the relative increase in elongation at break of PLA/PBS blends using different types and contents of compatibilizers, including low molecular weight compatibilizers, inorganic fillers, homopolymers, and copolymers.

Upon confirming the compatibilization effect of ML‐MOF nanoparticles on the immiscible PLA/PBS blend, we examined the resulting changes in the blend's mechanical properties. The stress‐strain curves of the PLA/PBS and PLA/PBS/ML‐MOF blend with varying ligand ratios are shown in **Figure** **3**a, with detailed data summarized in Table S2 (Supporting Information). The PLA/PBS blend exhibits a typical brittle fracture pattern. In contrast, the blends incorporating ML‐MOF nanoparticles demonstrate ductile behavior, sustaining comparatively larger strains before fracturing. The distinct difference illustrates the reinforcement effect of ML‐MOF nanoparticles. Furthermore, the variation in the stress‐strain curves indicates different degrees of reinforcement, which can be attributed to the spatial arrangement of ML‐MOF nanoparticles in the blend, as dictated by the ligand ratio.

Figure 3b presents the specific mechanical properties extracted from the stress‐strain curves. The addition of ML‐MOF_(5:5)_ nanoparticle resulted in significant reinforcement of the blends, with tensile strength increasing from 48.1 to 53.3 MPa, Young's modulus rising from 944.5 to 1096.1 MPa, and the tensile toughness improving from 3.1 to 104.0 J cm^−3^. Furthermore, the elongation at break dramatically increased from 9.1% to 229.2%, demonstrating a remarkable enhancement in ductility. Other amphiphilic ratios also improved the mechanical properties: for ML‐MOF_(2:8)_ and ML‐MOF_(8:2)_ nanoparticles, the elongation at break increased to 123.6 and 157.7%, Young's modulus increased to 1047.2 and 1060.2 MPa, tensile strength increased to 52.1 and 50.4 MPa, and tensile toughness increased to 45.1 and 50.4 J cm^−3^, respectively. Although the mechanical properties of PLA/PBS/ML‐MOF_(0:10)_ and PLA/PBS/ML‐MOF _(10:0)_ blends were higher than those of the neat PLA/PBS blend, the enhancements were relatively smaller compared to the blends containing other amphiphilic nanoparticles.

Alongside the experimental investigation, we utilized MD simulations to further explore the underlying reinforcement mechanism imparted by ML‐MOF nanoparticles. Uniaxial deformation was performed on the glass state model systems shown in Figure 1a, simulating the void generation inside the system by fixing the box size perpendicular to the elongation direction according to the experimental operating temperature. Figure 3c presents the yielded stress‐strain curves. Figure 3c presents the yielded stress‐strain curves. Although the simulation results, due to model simplicity (e.g., generic force field, non‐breakable polymer bonds, and limited size), do not depict abrupt fracture or brittle‐ductile transitions as observed in the experiment, the progression of the stress‐strain curves captures the qualitative behavior shown in Figure 3a. Additionally, the variations in extracted yield strength and Young's modulus from the simulations show good agreement with the experimental data in Figure 3b, confirming the consistency between simulation and experimental observations.

Furthermore, we conducted a comprehensive comparison of the results of this study with those of other recently published studies. The PLA/PBS blend containing ML‐MOF_(5:5)_ nanoparticle exhibited a remarkable over 30‐fold increase in elongation at break, despite the mere 0.1 wt.% filler content (Figure 3e). Details of the compatibilizers used for comparison and their sources are listed in Table S3. Compared to other compatibilizers described in the literature, this represents a significant improvement. The pronounced enhancements in mechanical properties suggest that ML‐MOF_(5:5)_ nanoparticle could serve as a leading‐edge compatibilizer for PLA/PBS blend. It is also noteworthy that, in addition to the superior compatibilization enhancement effect, this strategy requires less stringent processing conditions compared to conventional copolymerization^[^ ^53^ ^]^ or in situ compatibilization enhancement strategies,^[^ ^54^ ^]^ and the process is simpler as it does not require pre‐modification of the compatibilizer.^[^ ^55^ ^]^ Notbaly, the actual mechanical performance enhancement observed after the introduction of ML‐MOF nanoparticles exceeded the predictions from preliminary simulations (Figure 3c,d), emphasizing the crucial role of ML‐MOF nanoparticles in promoting compatibility within the blend system.

**Figure 4:**
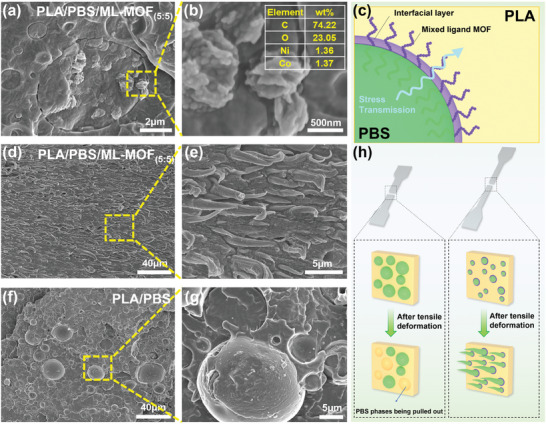
Compatibilization mechanism of ML‐MOF_(5:5)_ nanoparticle in PLA/PBS blend: a) Cross‐sectional surface of PLA/PBS/ML‐MOF_(5:5)_ and b) Elemental composition of PLA/PBS/ML‐MOF_(5:5)_ blend. c) Schematic illustration of the compatibilization mechanism of ML‐MOF_(5:5)_ nanoparticle. d, e) Tensile fracture surface features of the PLA/PBS/ML‐MOF_(5:5)_ blend. f,g) Tensile fracture surface features of the PLA/PBS blend. h) Schematic illustration of the morphological evolution of the PBS dispersed phase during the tensile deformation of PLA/PBS and PLA/PBS/ML‐MOF_(5:5)_ blends.

Prompted by the enhanced mechanical properties of the blends with just 0.1 wt.% ML‐MOF_(5:5)_ nanoparticle, we conducted a detailed examination of the cross‐section and tensile fracture morphology. Comparison of the interfacial morphology of the blends (**Figure** **4**a; Figure S17, Supporting Information) showed that the integration of 0.1 wt.% ML‐MOF_(5:5)_ nanoparticle predominantly localized at the interface between the PLA and PBS phases. This interface analysis using EDS confirmed a Ni–Co ratio of ≈1:1 (Figure 4b), indicating the presence of ML‐MOF_(5:5)_ nanoparticles. Figure 4c illustrates the positioning of ML‐MOF(5:5) nanoparticles at the PLA/PBS blend interface, highlighting their role in enhancing the blend's compatibility. Notably, the PBS dispersed phases under external forces exhibited different morphologies between PLA/PBS and PLA/PBS/ML‐MOF_(5:5)_ blends. Further investigation into the tensile fracture morphology (Figure 4d–g) revealed that, in the absence of ML‐MOF_(5:5)_, the PLA/PBS blend displayed brittle fractures characterized by a pronounced gap between the PBS and PLA phases, indicating poor compatibility and a fragile interface that facilitated the detachment of the PBS phase from the PLA phase under external pressure. In contrast, with the incorporation of ML‐MOF_(5:5)_ nanoparticles, a significant improvement in compatibility was observed. The interphase gap between PBS and PLA diminished, and under tensile force, the PBS phase morphed into root‐like fibers rather than detaching directly.

Overall, the improved compatibility and mechanical properties of PLA/PBS blends can be attributed to four pivotal factors. First, the amino functional group in the ML‐MOF_(5:5)_ nanoparticle's organic ligand forms hydrogen bonds with the carbonyl group in the PBS phase.^[^ ^56^, ^57^ ^]^ This interaction significantly enhances PLA‐PBS compatibility by facilitating stress transfer at the interface, allowing PBS to form fibrous structures when tensile force is applied, thereby dissipating energy and increasing the tensile toughness of the blend (Figure 4h).^[^ ^31^ ^]^ Second, during the blending process, the polymer chains of the two phases may become entangled in the pores of ML‐MOF nanoparticles, creating a mechanical interlocking structure.^[^ ^58^, ^59^ ^]^ This interlocking not only tightly bonds the two‐phase polymers but also prevents microphase separation, thereby reinforcing the material's structural integrity. Third, the ML‐MOF_(5:5)_ nanoparticle lowers interfacial tension and increases interfacial adhesion by depositing at the interface, enhancing cohesion between the phases. Finally, the addition of ML‐MOF_(5:5)_ nanoparticle increases the viscosity of PLA matrix (Figure S18, Supporting Information), which modulates the melting dynamics of the dispersed PBS phase and inhibits phase separation.^[^ ^60^ ^]^ This combined effect of molecular interactions and phase dynamics contributes to the overall improved mechanical properties of polymer blends.

### 2.4 Universality of ML‐MOF Compatibilization‐Enhancing Effects in Various Polymer Blends

**Figure 5:**
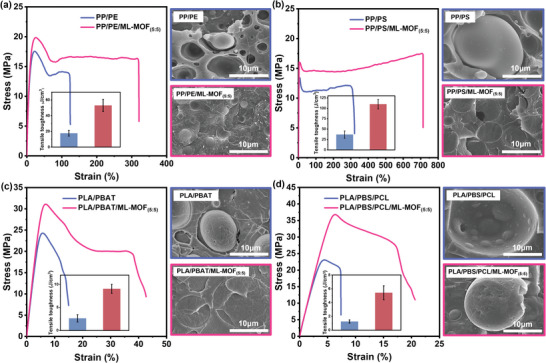
Demonstrating the universality of compatibilization‐enhancing effects: a) Comparison of stress‐strain curves and tensile toughness between PP/PE and PP/PE/ML‐MOF_(5:5)_, along with their cryofracture surfaces. b) Stress‐strain curves and tensile toughness for PP/PS and PP/PS/ML‐MOF_(5:5),_ with corresponding cryofracture surfaces. c) Stress‐strain curves and tensile toughness of PLA/PBAT versus PLA/PBAT/ML‐MOF_(5:5)_, including images of their cryofracture surfaces. d) Stress‐strain curves and tensile toughness of PLA/PBS/PCL compared to PLA/PBS/PCL/ML‐MOF_(5:5)_, with corresponding cryofracture surface images.

To assess the broad applicability of the ML‐MOF nanoparticle compatibilization strategy for strengthening and toughening different polymer blends, we first investigated its effects on a polyolefin blend of polypropylene and polyethylene (PP/PE, 70/30 w/w). **Figure** **5** displays the tensile stress‐strain curves and corresponding SEM images illustrating the fracture morphology of various polymer blends. As depicted in Figure 5a, the incorporation of 0.1 wt.% ML‐MOF_(5:5)_ into the PP/PE blend increased the elongation at break from 124.4% to 321.0% and the tensile toughness from 17.7 to 53.0 J cm^−3^, while the tensile strength increased from 17.5 to 18.4 MPa. We then analyzed a blend of polyolefin with a polar plastic, specifically PP/polystyrene (PS) (70/30, w/w), as shown in Figure 5b. The addition of 0.1 wt.% ML‐MOF_(5:5)_ nanoparticle significantly improved the compatibility of the PP/PS blend, increasing the elongation at break from 321.7% to 713.9% and tensile toughness from 36.8 to 109.9 J cm^−3^, while the tensile strength increased from 13.4 to 15.9 MPa. Building on the results from the PLA/PBS system, we extended our research to other biodegradable polymer blends, such as PLA/PBAT (70/30, w/w) and a ternary blend of PLA/PBS/PCL (70/20/10, w/w). The addition of 0.1 wt.% ML‐MOF_(5:5)_ nanoparticle to the PLA/PBAT blend enhanced the elongation at the break by 284.0%, from 15.0% to 42.7% (Figure 5c), and showed a significant increase in tensile strength from 24.3 to 31.1 MPa. In the PLA/PBS/PCL blend, the addition of ML‐MOF_(5:5)_ nanoparticle markedly altered the tensile strength of the blend from 22.1 to 36.8 MPa and elongation at break from 7.3% to 20.6%, along with a dramatic increase in tensile toughness (Figure 5d). In binary blending systems, the addition of ML‐MOF nanoparticles reduced the dispersed phase size, with the amphiphilic nature of the MOF causing the nanoparticles to remain at the interface, thereby improving interface strength and facilitating stress transfer between the phases.^[^ ^61^ ^]^ In a ternary blending system, the changes in mechanical properties, particularly tensile strength, are pronounced due to the reduced accumulation of PCL around the PBS phase, as ML‐MOF nanoparticles tend to stay at the PLA‐PBS interface.^[^ ^62^, ^63^ ^]^ Under external forces, stress was transferred from the PLA to PBS or PCL phase. However, due to the relatively low interfacial strength between PCL and PLA phases, stress transfer is more difficult, resulting in a smaller increase in fracture elongation but a significant increase in tensile strength. Based on this analysis, it is likely that ML‐MOF nanoparticles in the multiphase blend system selectively disperse at the interface between phases with strong nanoparticle affinity, acting as a “zipper” to increase interfacial strength. Additionally, when an external force is applied, the ML‐MOF nanoparticle acts as a “bridge,” facilitating stress transfer from the hard phase to the soft phase, thereby absorbing energy and reducing the likelihood of material failure. In summary, the increased strength and toughness of these blends illustrate the ability of ML‐MOF nanoparticles to significantly improve the mechanical properties of various polymer blends, underscoring the broad applicability of this compatibilization strategy.

### 2.5 Potential Application of PLA/PBS/ML‐MOF Blend in Food Packaging

**Figure 6:**
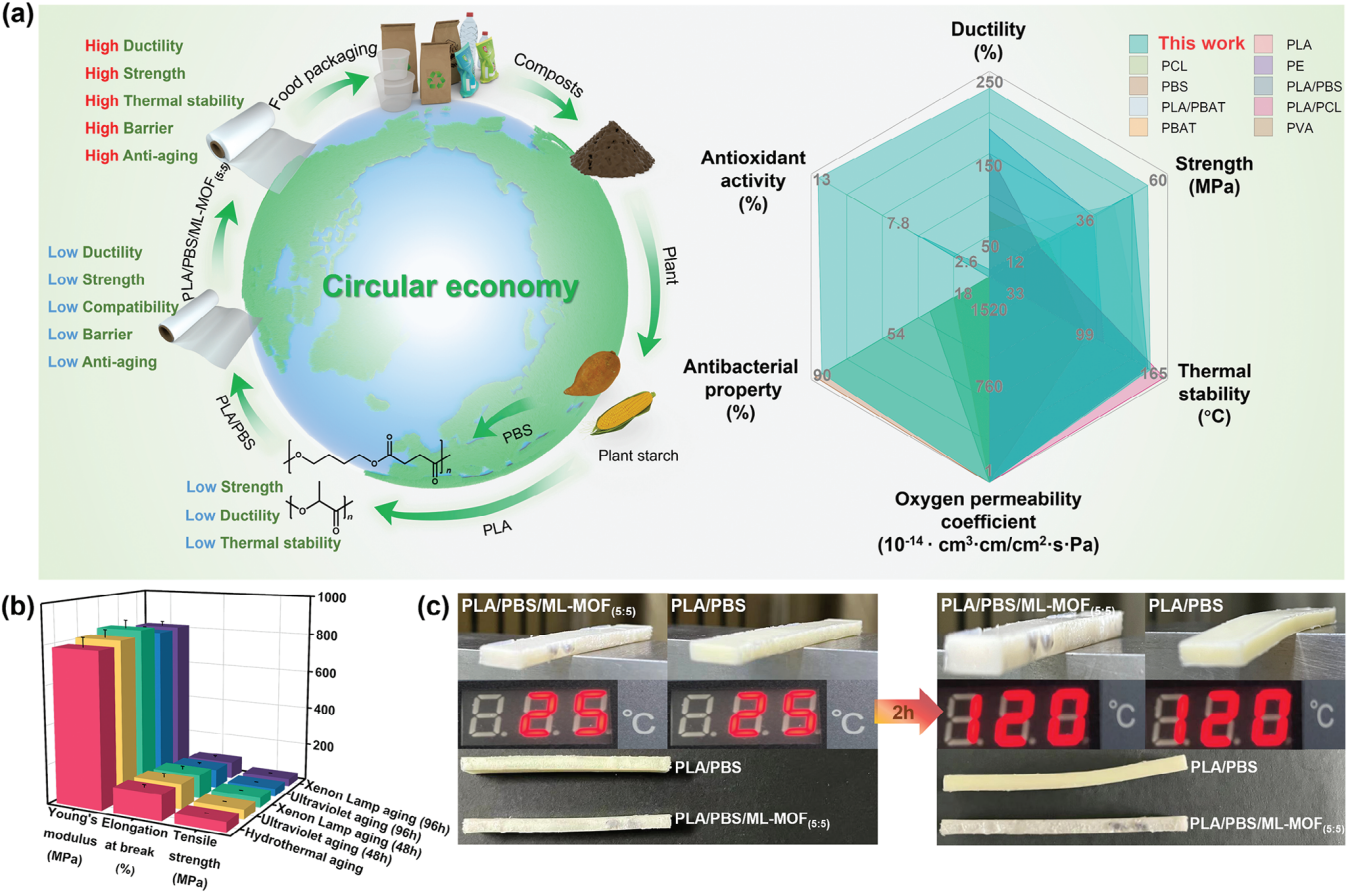
Potential applications of the PLA/PBS/ML‐MOFs blend film: a) Left: Fabricated PLA/PBS/ML‐MOF_(5:5)_ film demonstrating excellent performance and biodegradability, suitable for food packaging applications. Right: Performance comparison between PLA/PBS/ML‐MOF_(5:5)_ and other commonly used packaging films. b) Mechanical properties of PLA/PBS/ML‐MOF_(5:5)_ blends after hydrothermal aging, xenon lamp aging, and UV aging. c) Deformation of PLA/PBS/ML‐MOF_(5:5)_ and PLA/PBS blends after heat treatment in an oven.

In this study, ML‐MOF_(5:5)_ nanoparticles were effectively incorporated into the PLA/PBS blend, resulting in materials with excellent mechanical properties, as well as significant improvements in heat resistance, aging resistance, antimicrobial activity, oxygen resistance, and antioxidant properties (Figures S19‐22). Crucially, the biodegradability of the base polymer were preserved despite these enhancements, ensuring that the PLA/PBS/ML‐MOF_(5:5)_ blend remains environmentally friendly and suitable for practical applications (Figure S23, Supporting Information). Building on the preserved biodegradability of both PLA and PBS, the PLA/PBS/ML‐MOF_(5:5)_ blend demonstrates promising potential for food packaging applications. Compared to conventional packaging films, this blend not only retains its eco‐friendly properties, but also offers enhanced ductility, tensile strength, heat resistance, and improved antioxidant and antibacterial functions, even at low filler content (**Figure** **6**a; Table S4, Supporting Information).

Moreover, the mechanical stability of the PLA/PBS/ML‐MOF_(5:5)_ blend was evaluated under various environmental conditions, including hydrothermal exposure, UV irradiation, and xenon lamp irradiation exposure (Figure 6b). Although a slight reduction in mechanical properties was observed with prolonged exposure, the blend maintained significantly higher tensile strength, elongation at break, and Young's modulus compared to the PLA/PBS blend, indicating its robust stability and suitability for real‐world applications. Furthermore, the thermal deformation behavior of these blends was assessed at 120 °C for 1 h before and after the addition of ML‐MOF_(5:5)_ nanoparticle (Figure 6c). Neat PLA/PBS blend tended to deform, whereas the incorporation of ML‐MOF_(5:5)_ nanoparticle resulted in reduced deformation and improved shape retention. This improvement was attributed to enhanced compatibility, which elevated the melting temperature (*T_m_*) and promoted heterogeneous nucleation of PLA and PBS phases, thereby increasing the crystallinity of both entities (Figure S19 and Table S5, Supporting Information). Thermogravimetric analysis (TGA, Figure S20 and Table S6, Supporting Information) indicated an improvement in the thermal stability of the PLA/PBS/ML‐MOF_(5:5)_ blend, with a 5% increase in the onset of degradation temperature (*T_d_*). The slight drop in *T_max_* might be due to the production of metal compounds during the thermal degradation of ML‐MOF_(5:5)_ nanoparticles, acting as catalysts that expedite the depolymerization of PLA, producing lactic acid and accelerating the thermal degradation of the PLA/PBS/ML‐MOF_(5:5)_ blend. Additionally, in the low‐frequency spectrum, both PLA/PBS/ML‐MOF and PLA/PBS blends exhibited viscous tendencies (tan δ > 1); however, with increasing frequency, the tan δ of PLA/PBS/ML‐MOF diminished, indicating solid characteristics and adaptability to processing under reduced shear forces (Figure S16, Supporting Information). Consequently, the PLA/PBS/ML‐MOF_(5:5)_ blend offers a synergistic portfolio of properties, demonstrating its potential for application in food packaging.

## 3 Conclusion

In summary, coarse‐grained molecular dynamics (MD) simulations revealed a volcano‐like dependency between the compatibilizing properties and the amphiphilicity of the compatibilizers. Building on these insights, ligand‐modulated MOF nanoparticles with tunable hydrophilicity and lipophilicity were developed as versatile compatibilizers to significantly enhance the toughness and strength of various polymer blends. Specifically, in the PLA/PBS blend, the incorporation of ML‐MOF nanoparticles with optimal amphiphilicity (hydrophilicity:lipophilicity = 5:5) led to approximately a 1.1‐fold increase in tensile strength and a 34.1‐fold increase in toughness compared to the neat PLA/PBS blend. This enhancement was attributed to the improved interfacial compatibility between PLA and PBS. Moreover, the effectiveness of ML‐MOF_(5:5)_ nanoparticles as universal compatibilizers was demonstrated in other high‐strength, high‐toughness polymer blends, including PP/PE, PP/PS, PLA/PBAT, and PLA/PCL/PBS. Furthermore, ML‐MOF_(5:5)_ nanoparticles conferred enhanced oxygen barrier properties, improved thermal resistance and stability, and antibacterial capabilities to the PLA/PBS blend, highlighting their significant potential for practical applications across a broad range of polymer blend systems.

## Conflict of Interest

The authors declare no conflict of interest.

## Author Contributions

T.K., W.L., W.X.L., and S.S.S. performed conceptualization. H.G. and W.G. conducted the methodology. H.G., W.G., and W.L. carried out the investigation and simulation. T.K., H.G., W.G., W.L., W.X.L., M.R.S., M.V., and S.S.S. performed the formal analysis. T.K., W.L., W.X.L., and S.S.S. provided supervision. H.G., W.L., W.X.L., and T.K. wrote the original draft. T.K., W.L., W.X.L., M.R.S., M.V., and S.S.S. reviewed and edited the draft.

## Supporting information



Supporting Information

## Data Availability

The data that support the findings of this study are available from the corresponding author upon reasonable request.
