# Supplementary material for: Boosting the Strength and Toughness of Polymer Blends via Ligand‐Modulated MOFs
Source: Adv Sci (Weinh). 2024 Oct 16;11(45):2407593. doi: 10.1002/advs.202407593 (PMC11615806; doi:10.1002/advs.202407593)
Supplement: Supplementary file 1 — Supporting Information [file ADVS-11-2407593-s001.docx]

**Supporting Information**

***Boosting the Strength and Toughness of Polymer Blends via*** ***Ligand-Modulated MOFs***

*Tairong Kuang*, Hongxin Guo, Wei Guo, Wenxian Liu*, Wei Li*, Mohammad Reza Saeb, Mohammad Vatankhah-Varnosfaderani, Sergei S. Sheiko**

**T. R. Kuang, H. X. Guo, W. Guo, W.X. Liu**

Functional Polymers & Advanced Materials (FPAM) Lab, Zhejiang Key Laboratory of Plastic Modification and Processing Technology, College of Materials Science and Engineering, Zhejiang University of Technology, Hangzhou 310014, P. R. China

**E-mail:** *kuangtr@zjut.edu.cn*, *liuwx@zjut.edu.cn*

**W. Li**

Institute for Chemical Reaction Design and Discovery (WPI-ICReDD), Hokkaido University, Sapporo 001-0021, Japan

Suzhou Laboratory, Suzhou, Jiangsu 215123, P. R. China

**E-mail:** *wel208@icredd.hokudai.ac.jp*

M. R. Saeb

Department of Pharmaceutical Chemistry, Medical University of Gdańsk, J. Hallera 107, 80-416 Gdańsk, Poland

M. Vatankhah-Varnosfaderani, S. S. Sheiko

Department of Chemistry, University of North Carolina at Chapel Hill, Chapel Hill, North Carolina 27599, United States

**E-mail:** *sergei@email.unc.edu*

Experimental Sections

### *Materials*

Poly(lactic acid) (PLA) pellets (4032D) were purchased from NatureWorks LLC (USA). The PLA pellets had a specific density of 1.24 g/cm^3^ and a melt flow index (MFI) of 6.17 g/10 min ((210 °C /2.16 kg)). The weight average molecular weight (M_w_) and number average molecular weight (Mn) of the PLA were determined to be 1.85×10^5^ g/mol and 1.22×10^5^ g/mol, respectively. Polybutylene succinate (PBS) pellets (TH801) were purchased from Xinjiang Lan Shan Tun He New Materials Co., Ltd. (China). The pellets possess a specific density of 1.26 g/cm^3^, an MFI of 4.5 g/10min, and a melting point range of 110-120 °C. Furthermore, M_w_ and M_n_ of the PBS pellets are determined to be 5.11×10^4^ g/mol and 2.83×10^4^ g/mol, respectively. Poly (butyleneadipate-co-terephthalate) (PBAT) pellets (Ecoflex-F-Blend-C1200) and polyethylene (PE) pellets (2420H) were sourced from BASF (Germany). Polypropylene (PP) pellets (E02ES) were obtained from Sinopec Zhenhai Refining & Chenical Company (China). Polycaprolactone (PCL) pellets (CAPA 6500) came from Perstorp Co., Ltd. (Sweden), and polystyrene (PS) pellets (GH-660) from China Petroleum & Chemical Corp (China).

The reagents used in the synthesis of mixed-ligand metal-organic framework (ML-MOF) nanoparticles were sourced as follows: nickel chloride hexahydrate (NiCl_2_-6H_2_O, ≥ 99.9%), terephthalic acid (BDC, 99%) and 2-amino-terephthalic acid (NH_2_-BDC) were purchased from was purchased from Beijing Bailingwei Technology Co. Ltd. (China). Cobalt chloride hexahydrate (CoCl_2_-6H_2_O, 99.9%) was obtained from Aladdin Chemistry Co., Ltd. (China). N, N-dimethylformamide (DMF, AR grade), triethylamine (TEA, AR grade), and ethanol (AR grade) were supplied by Shanghai Sinopharm Chemical Reagent Co., Ltd. (China). Ultrapure water was prepared using a HHitech (RO DI) water purification system.

### *Coarse-Grained Molecular Dynamics Simulations*

We adopted coarse-grained molecular dynamics (MD) simulations to explore the interfacial behaviors influenced by amphiphilic nanoparticles (NPs) in a binary blend of immiscible polymer A (pA) and polymer B (pB). The immiscibility is dictated by the repulsion between pA and pB monomers (Table S1). In the model system, the phase separation leads to an isolated B phase domain inside the A polymer matrix (Fig. S1). The amphiphilic NP comprises two types of organic affinity units: oA and oB. The oA unit is attractive to pA and repulsive to pB, while oB has a preferential attraction with pB but a repulsion with pA. Tuning the relative quantities of oA and oB adjusts the NP’s overall amphiphilicity, thereby dictating its affinity with different types of polymers. Five number ratios of oA and oB affinity units are examined in this work, i.e., ${n_{\mathrm{oA}}:n}_{\mathrm{oB}}=10:0$, $8:2$, $5:5$, $2:8$, and $0:10$ (Fig. S1). Accordingly, the nanoparticle systematically changes from purely attractive to A polymers to purely attractive to B polymers, characterized by the overall fraction of oB affinity units, $f_{\mathrm{oB}}$. Through MD simulations performed by LAMMPS, we examined the phase behavior and mechanical properties of the mixture systems, exposing the effects of amphiphilic nanoparticles as compatibilizing and reinforcing agents of the polymer blend. A detailed description of the model and MD simulation protocol is presented as follows:

To investigate the interfacial behaviors of incompatible polymer blends, a coarse-grained (CG) model was developed, simulating a binary blend of immiscible linear A and B homopolymers along with amphiphilic nanoparticles (NPs). Standard Lennard–Jones (LJ) reduced units, with unit length $\sigma$, energy $\epsilon$, mass $m$, and time $\tau=\sqrt{m\sigma^{2}/\epsilon}$, were employed in the model. The homopolymers are represented by a bead-spring chain, which consists of $N=40$ pA or pB beads, and due to their immiscibility, phase separation takes place in the neat polymer blend (Fig. S1a). Each monomer bead has a diameter of $\sigma$, and the adjacent beads along the chain contour are connected by a FENE bond potential, $U_{\mathrm{FENE}}\left( r \right)=-0.5k_{b}R_{0}^{2}\ln\left( 1-r^{2}/R_{0}^{2} \right)$, where the spring constant $k_{b}=30\epsilon/\sigma^{2}$ and the maximum length of $R_{0}=1.5\sigma$. The NP is modeled by a rigid spherical shell of a diameter $5\sigma$, comprising 226 beads (each of diameter $\sigma$) arranged uniformly on the shell (Fig. S1b). The bead identity is randomly assigned as oA or oB according to their total number ratio $n_{\mathrm{oA}}:n_{\mathrm{oB}}$, while oA has an attraction with pA but a repulsion with pB and vice versa for the interactions of oB with pA and pB (Table S1). The mixture system, if not otherwise specified, contains $n_{A}=180$ A chains, $n_{B}=3600$ B chains, and $n_{\mathrm{NP}}=20$ NPs in a cubic simulation box. Periodic boundary conditions are applied in all three directions. This setting ensures the investigation of interfacial behaviors in a phase-segregated system with a manageable computational demand.


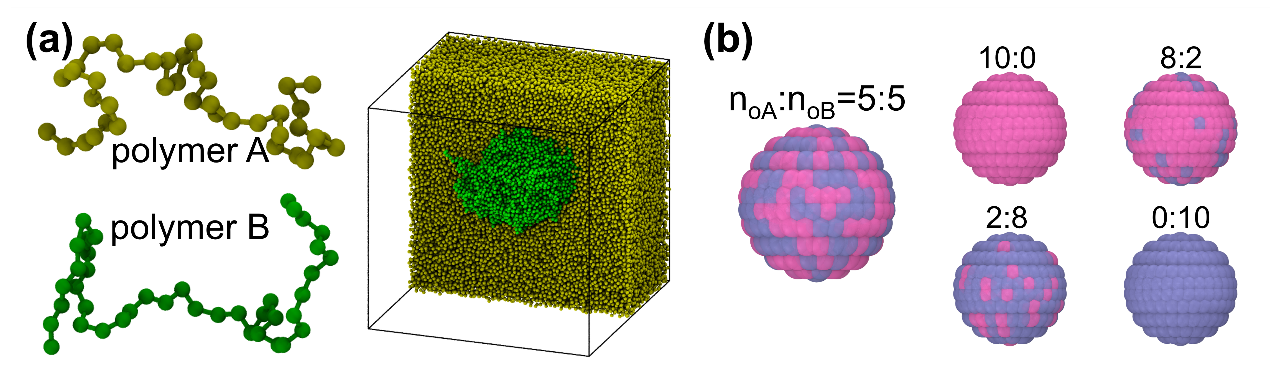


**Fig. S1.** System setup and composition of the polymer blend and amphiphilic nanoparticles used in the study: (a) System setup of A and B homopolymers (both of chain length $N=40$) and phase-separated blend (with $n_{A}=180$ A polymers and $n_{B}=3600$ B polymers). In the blend, B polymers form an isolated domain inside A polymer matrix. Only half of the A beads are shown in the cubic simulation box for visualization. (b) Amphiphilic nanoparticles are composed of two types of organic affinity units, namely, oA and oB, at different number ratios ${n_{\mathrm{oA}}:n}_{\mathrm{oB}}$. The total unit number is fixed at $n_{o}=n_{\mathrm{oA}}+n_{\mathrm{oB}}=226$.

A generic force field was invoked for the model system to study the general phase behavior and the reinforcement effect of NPs. The repulsion is depicted by a purely repulsive Weeks–Chandler–Andersen (WCA) potential:

$$U_{\mathrm{WCA}}\left( r_{ij} \right)=\left\{ \begin{aligned} 4\varepsilon_{\mathrm{WCA}}\left[ \left( \frac{\sigma}{r_{ij}} \right)^{12}-\left( \frac{\sigma}{r_{ij}} \right)^{6}+\frac{1}{4} \right], r_{ij}\leq2^{1/6}\sigma\\ 0, r_{ij}>2^{1/6}\sigma\end{aligned} \right.$$

where $i$ and $j$ are indices of different beads. The attraction is represented by standard LJ potential, $U_{\mathrm{LJ}}\left( r_{ij} \right)=4\varepsilon_{\mathrm{LJ}}\left[ \left( \frac{\sigma}{r_{ij}} \right)^{12}-\left( \frac{\sigma}{r_{ij}} \right)^{6} \right]$, with a cutoff applied at $2.5\sigma$. In this study, we used molecular dynamics (MD) simulations to separately examine the phase segregation of the mixture in the melt state and the effects of NPs on the mechanical properties in the glass state. Accordingly, two typical sets of force fields were employed (Table S1), with the system temperature fixed at $T=1.5$ and $0.4$ and external pressures at $P=5.0$ and $0.0$ for the melt and glass states, respectively. We note that by combining the associated force field and pressure settings, the model systems essentially capture the same melt state of the polymer blend at $T=1.0$, with an overall number density of $\rho\approx0.85$. However, using mainly repulsions in the force field facilitates the phase separation process during the temporal evolution.

For the initial configuration, the polymer chains and NPs were inserted in a large cubic box (with a side length of 600σ). The starting beads of individual polymer chain propagation and nanoparticle generation were randomly distributed inside the box while being far away from each other (with separation larger than 10σ) to avoid overlapping. For phase-separated systems, the minority chains were initialized inside a shell (of a diameter 100σ) centered at the box center to facilitate the kinetic process of segregation. After relaxation by energy minimization, the system was evolved in the NPT ensemble. The timestep size is fixed at $\Delta t=0.01\tau$. A Langevin thermostat was adopted to control the system temperature, with a damping parameter of $100\tau$. The system was first prepared in the melt state. The equilibration was run for more than $2\times{10}^{7}$ time steps, after which the system reached a long-lasting steady state. Then, a production run was conducted to generate a trajectory of $5\times{10}^{6}$ time steps for the analysis of morphological properties.

From the melt state, the glass state was prepared by switching the force field and external pressure setting and quenching the temperature directly from $T=1.5$ to $T=0.4$. The equilibration was run for $1\times{10}^{7}$ time steps. The final configuration was taken as an input state for a subsequent uniaxial extension run (along the $x$-axis) at a constant strain rate of $\dot{\varepsilon}={10}^{-4}$ for $5\times{10}^{6}$ time steps. During the extension, the simulation box was fixed in the $y$ and $z$ directions which allowed for the void generation and complete rupture. From the stress-strain curve, the yield strength was extracted at the yield point, and Young’s modulus was determined from the slope in the initial linear region. All MD simulations were performed using LAMMPS. Statistical results were obtained by averaging over 5 independent runs of individual systems started with different initial configurations.

### *Synthesis of mixed-ligand metal-organic framework (ML-MOF) nanoparticles*

The synthesis of ML-MOF nanoparticles with different ligand ratios followed the same steps and conditions as described in the following steps, with only the molar ratio of BDC and NH_2_-BDC being varied. The total molar mass of BDC and NH_2_-BDC was maintained at 0.6 mmol. In this study, five ML-MOF were synthesized with different ligand ratios: BDC : NH_2_-BDC ratios of 0:10, 2:8, 5:5, 8:2, and 10:0. These variations were explored to determine the optimal ligand ratio for achieving the desired compatibilization effect.

Typically, ML-MOF nanoparticles with a BDC : NH_2_-BDC ratio of 5:5 were synthesized under the following conditions: NiCl_2_-6H_2_O (0.3 mmol), CoCl_2_-6H_2_O (0.3 mmol), BDC (0.3 mmol), and NH_2_-BDC (0.3 mmol) were dissolved in a mixed solution containing 32 ml of DMF, 2 ml of ethanol, and 2 ml of deionized water. This solution was added dropwise to the reaction mixture under continuous stirring. After the addition, 0.8 ml of TEA was introduced, and the mixture was ultrasonicated in an ultrasonic bath at room temperature for 1 h. Following sonication, the solution was placed on a magnetic stirring stage and stir continuously at room temperature for 5.5 h at a stirring speed of 400 rpm. The resulting mixture was then centrifuged, and the precipitate was washed three times with ethanol. Finally, the washed sample was dried in a vacuum oven at 60°C for 12 h, yielding a mauve powder.

### *Preparation of PLA/PBS/ML-MOF blend*

Prior to preparing the PLA/PBS/ML-MOF blend, both PLA and PBS pellets were dried in a vacuum oven at 60°C for 24 h to remove moisture. The PLA/PBS/ML-MOF blends were fabricated with a weight ratio of 70/30/0.1 (*w*/*w*/*w*). The blending process was conducted by directly melt blending the components at 180°C in a torque rheometer (Haake Polylab, QC) with a screw speed of 60 rpm for 8 min. After blending, the sample was compression molded under 180°C and 10 MPa for 3 min. To differentiate the blends with different ligand ratios, the nomenclature PLA/PBS/ML-MOF**_(_*_x_*_:_*_y_*_)_** was adopted, where *x* represents the molar ratio of BDC and *y* represents the molar ratio of NH_2_-BDC. For comparison, a control sample of the neat PLA/PBS blend (70/30, *w*/*w*) without ML-MOF was prepared using the same aforementioned steps.

### *Characterization*

The morphologies of ML-MOF were characterized by scanning electron microscopy (SEM, HITACHI Regulus 8100, HITACHI, Japan) and transmission electron microscopy (TEM, Tecnai G2 F30 Stwin，Philips-FEI，Netherlands). The Oxford Aztec series X-ray spectrometer was used to analyze the elemental composition of the MOF by area sweep. The mechanical properties of both PLA/PBS and PLA/PBS/ML-MOF blends were determined using an Instron 5966 mechanical testing apparatus (USA). The tensile samples had a thickness of 1 mm, and the tests were conducted at a crosshead speed of 10 mm/min. The fracture surface morphology and tensile fracture surfaces of both neat and PLA/PBS/ML-MOF_(5:5)_ samples were observed using a field emission electron scanning microscope (FE-SEM) (HITACHI Regulus 8100, HITACHI, Japan). The dispersion and size of the dispersed phase in the blends were examined using transmission electron microscopy (TEM) (HT7820, HITACHI, Japan). Contact angle measurements were conducted using a Dataphysics OCA20 instrument (Germany). The modulus distribution of the samples was analyzed using atomic force microscopy (AFM) (Bruker Multimode 8, Bruker, Germany). Heat resistance was assessed in a blast oven (DHG-6146A, Shanghai Jinghong Equipment Co., Ltd., China). Hydrothermal aging was conducted by heating the samples in an oil bath maintained at a constant temperature of 50°C. UV and Xenon aging tests were conducted using a Xenon lamp aging chamber (Atlas CI4000, Atlas, USA) and a UV-accelerated aging chamber (ZN-P, Zhongtian), respectively. The UV aging test was performed with an irradiation intensity of 60 W/m² for different times, while the Xenon lamp aging test was conducted with an irradiation intensity of 550 W/m² for different times.The absorbance at 517 nm was measured using a UV spectrophotometer to evaluate antioxidant performance. Free radical scavenging activity was calculated as follows: free radical scavenging activity (%) = (1-(A sample/A reference)) × 100. For antibacterial performance evaluation, E. coli was chosen as the test organism. The samples were incubated in the bacterial solution for 24 hours, and the Live/Dead BackLight kit (purchased from Thermo Fisher Scientific) was used to stain the bacteria. The characterization of the live and dead bacteria was performed using an inverted fluorescence microscope (Axio Observer. A1, Carl Zeiss, Germany) at a magnification of 40x (75 μm). Oxygen permeability was measured using an oxygen permeability meter (MOCON 221MD, MOCON, USA). Zeta potential measurements were conducted using dynamic light scattering (DLS) (Brookheaven Omnis, USA) to analyze the differences in activation energy among various ML-MOF surfaces. The test solution had a concentration of 1 mg/mL, using ethanol as the solvent. The NNLS algorithm was employed for the test, with a scattering angle of 90°. X-ray diffraction (XRD) (X’ Pert Pro, PANalytical, The Netherlands) was utilized for the structural characterization of the synthesized ML-MOF. The scanning speed is 5 °/min, and the scanning angle range is 5 to 50°. Fourier transform infrared resonance (FTIR) analysis was conducted on an FTIR spectrometer (Nicolet iS50, Thermo Fisher, USA) in the reflectance (ATR) mode, covering the range from 4000 to 400 cm^-1^. X-ray photoelectron spectroscopy (XPS) was performed using a K-Alpha instrument (Thermo Scientific, USA). The Raman spectra were collected using an HR Evolution Raman spectrometer (Horiba LabRAM, Japan) with a 633 nm laser. The measured wavenumber range was 4000 to 450 cm⁻¹. Dynamic mechanical analysis (DMA) was performed on all samples using a dynamic mechanical analyzer (Q800, TA Instruments, USA) operating in a single cantilever mode. The heating rate was set at 3°C/min with a frequency of 5 Hz and an amplitude of 15 μm. Differential scanning calorimetry (DSC) (Mettler-Toledo, Switzerland) was employed to evaluate the melting behavior of all samples. The samples were heated under a nitrogen atmosphere at a rate of 10°C/min from room temperature to 200°C. Rheological properties of the samples were tested using an advanced expanded rheometer (MCR 302, Anton Paar, Australia). Thermogravimetric analysis of PLA/PBS and PLA/PBS/ML-MOF_(5:5)_ blends were performed under nitrogen atmosphere by Thermogravimetric Analyzer (Discovery TGA55, TA Instruments, USA). The samples are heated from 25°C to 600 °C with the heating rate of 10 °C /min, and the curves of thermal gravity and thermogravimetric differential (DTG) during heating process are recorded. In the environmental friendliness assessment experiments, PLA/PBS and PLA/PBS/ML-MOF_(5:5)_ blends (10 mm × 10 mm × 1 mm) were immersed in 3 ml of PBS buffer (pH 7.2) at 60°C. The PBS buffer was replaced every 48 h. After each removal, the samples were rinsed with deionized water, thoroughly dried at 50°C, and then weighed. The weight loss rate during degradation was calculated using the following formula: W_loss_ (%) = (W_before_-W_after_)/W_before_×100, based on the sample weights before and after degradation. Using Origin software to create data graphs.

### Supplementary Results and Discussions


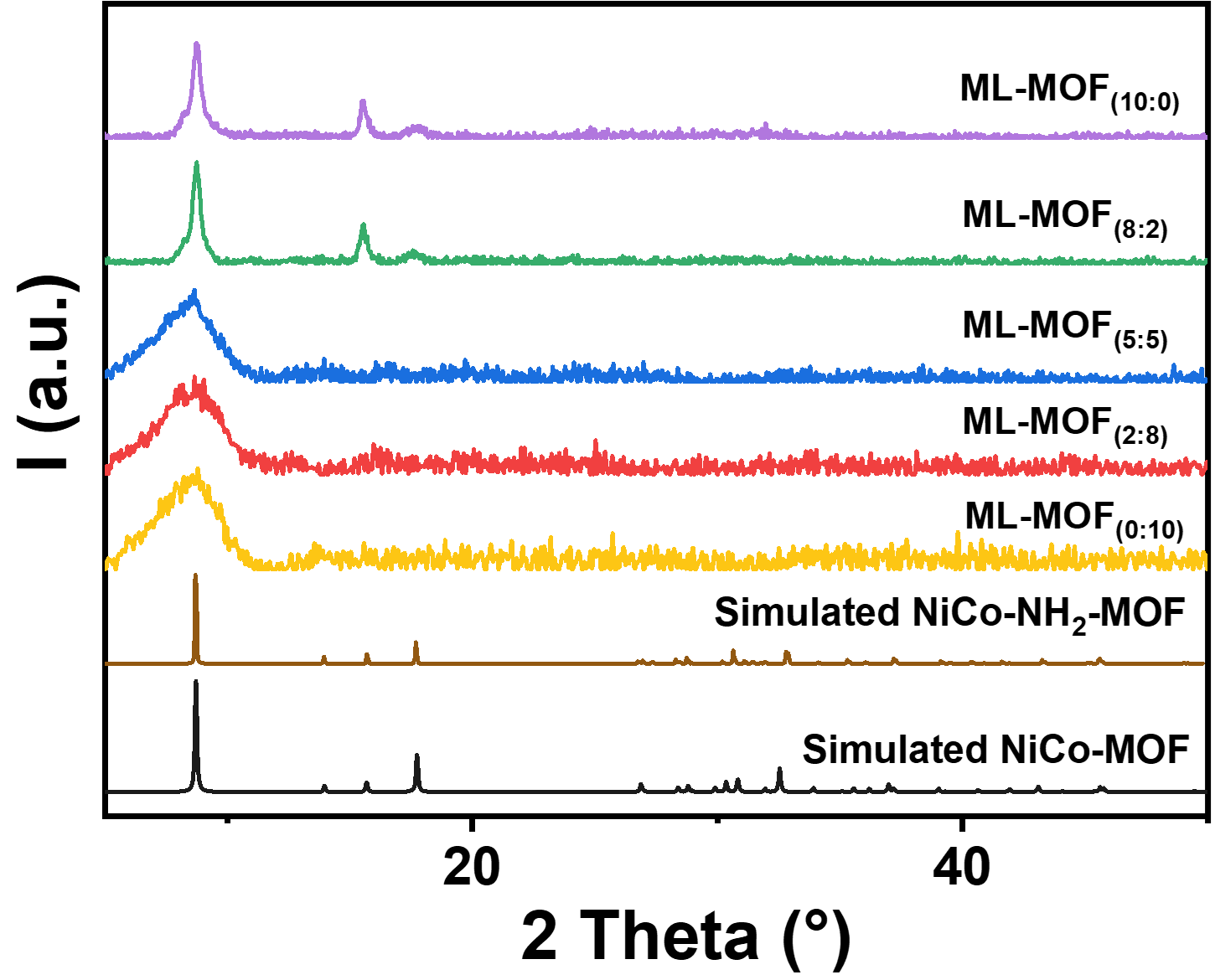


**Fig. S2.** XRD patterns of ML-MOF nanoparticles with varying ligand ratios of BDC to NH_2_-BDC.

Fig. S2 shows that ML-MOF nanoparticles with varying BDC to NH_2_-BDC ligand ratios exhibit distinct XRD patterns. Specifically, nanoparticles with ligand ratios of 10:0 and 8:2 show diffraction peaks at 2θ values of 8.9°, 15.6°, and 17.7°, corresponding to the (200), (311), and (321) planes of NiCo-MOF and NiCo-NH_2_-MOF respectively.^[1]^ Furthermore, ML-MOF nanoparticles with ligand ratios of 5:5, 2:8, and 0:10 exhibit a peak at 8.9°, corresponding to the (200) plane of NiCo-MOF and NiCo-NH_2_-MOF.


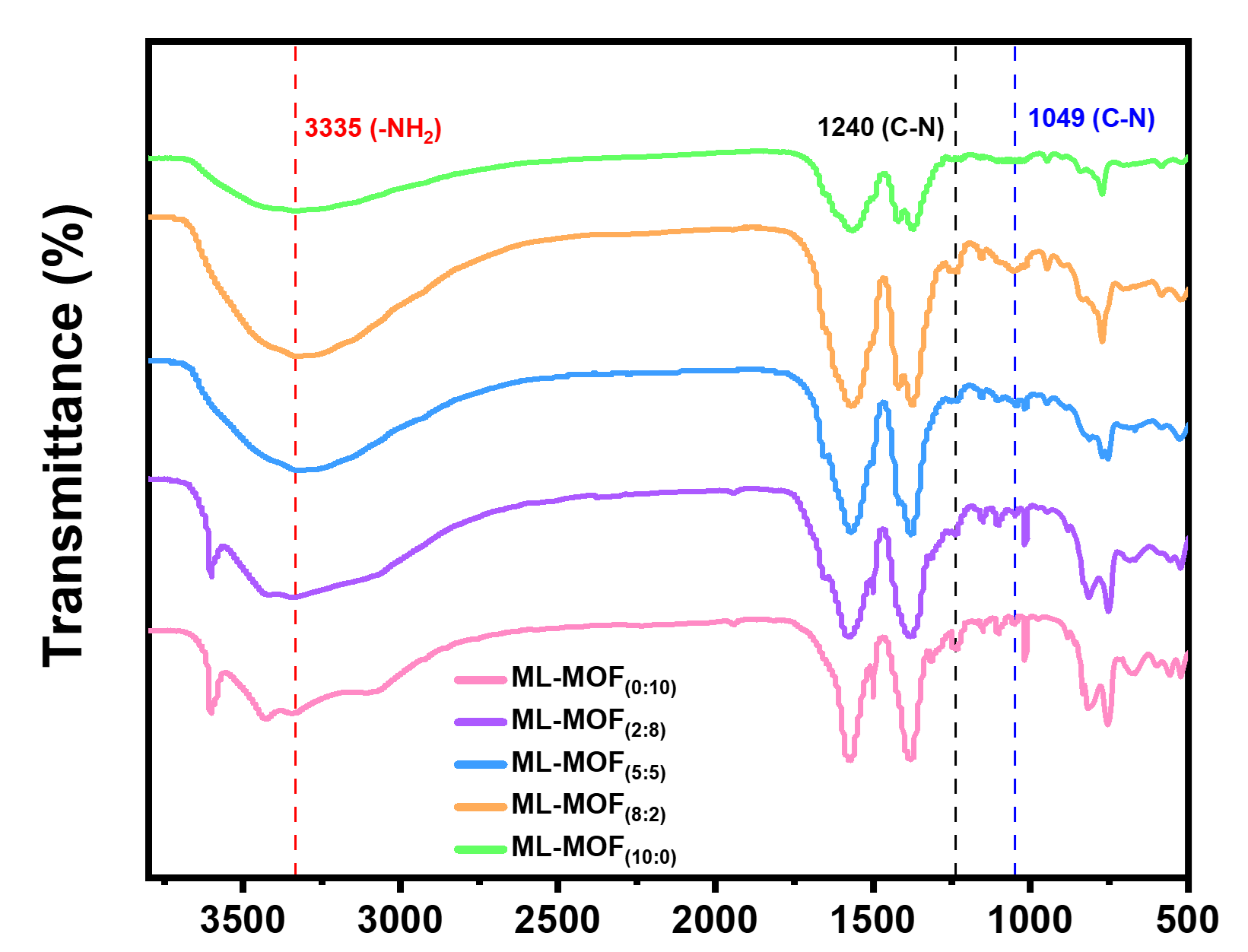


**Fig. S3.** FTIR spectra of ML-MOF nanoparticles with varying BDC/NH_2_-BDC ligand ratios.

Fig. S3 illustrates the FTIR spectra of ML-MOF nanoparticles with varying BDC/NH_2_-BDC ligand ratios. The spectra display characteristic absorption peaks at 1049 cm^-1^ and 1240 cm^-1^, associated with the C-N bond, and a peak at 3335 cm^-1^ corresponding to the stretching vibrations of the NH_2_ group. Notably, the intensity of the NH_2_ vibration peaks increases with a higher NH_2_-BDC ratio, indicating the successful incorporation of NH_2_-BDC ligands and their influence on the nanoparticle structure.


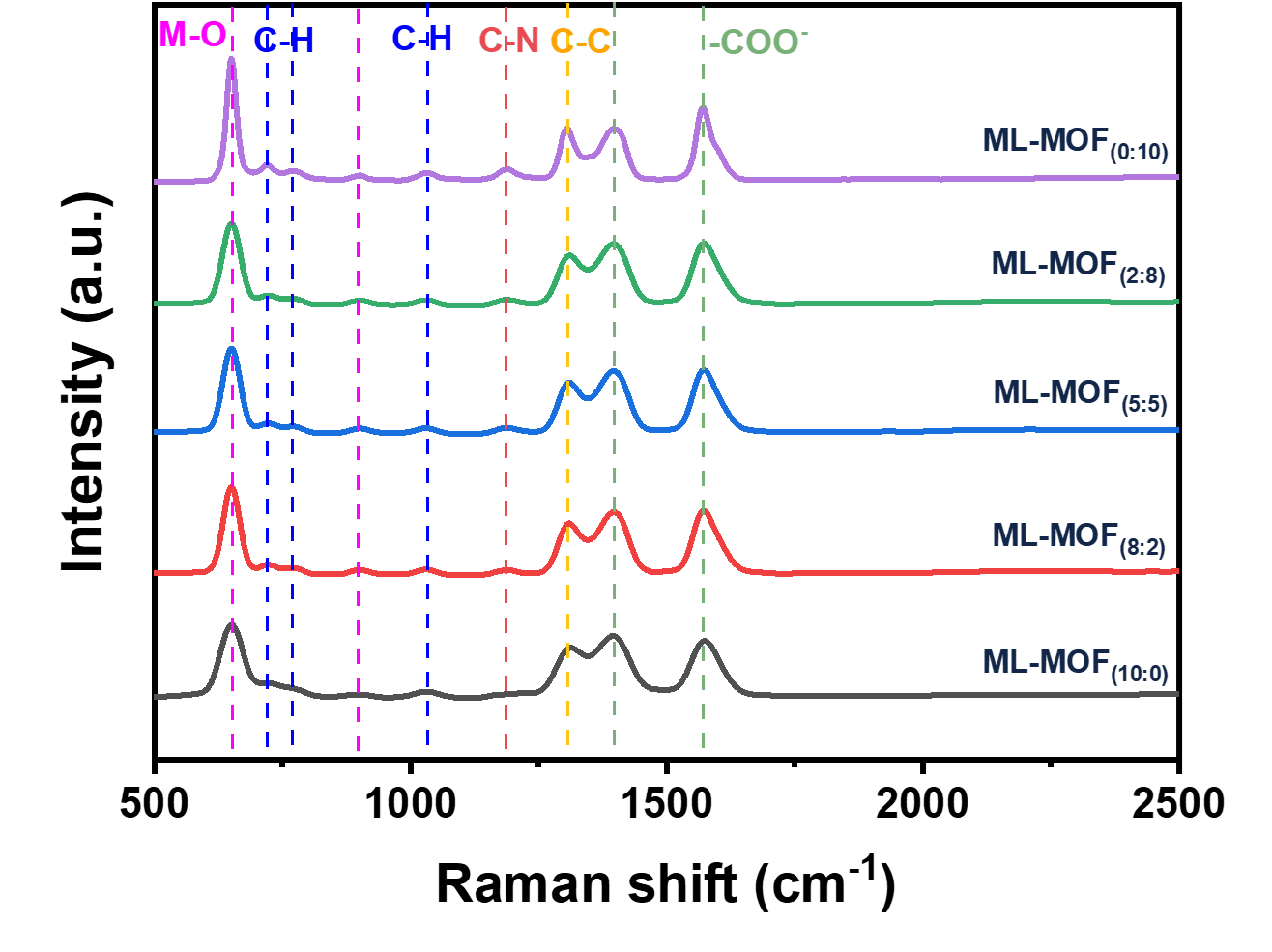


**Fig. S4.** Raman spectra of ML-MOF nanoparticles with different BDC/NH_2_-BDC ligand ratios.

Fig. S4 presents the Raman spectra of ML-MOF nanoparticles with different BDC/NH_2_-BDC ligand ratios. The peaks at 720, 769, 1030 and 1310 cm^-1^ correspond to the C-H and C-C vibrations of the benzene ring, ^[2-4]^ while the peaks at 1395 and 1570 cm^-1^ are attributed to the -COO^-^ stretching vibrations of the organic ligands coordinated to the metal.^[5-6]^ Additionally, the peaks at 654 and 890 cm^-1^ are assigned to the telescopic vibrations and asymmetric vibrations of the metal-oxygen bond, respectively.^[7-8]^ Notably, the Raman peak for C-N vibration^[9]^ at 1187 cm^-1^ increased progressively from ML-MOF_(10:0)_ to ML-MOF_(0:10)_, confirming the increasing proporation of NH_2_-BDC ligands in the samples.


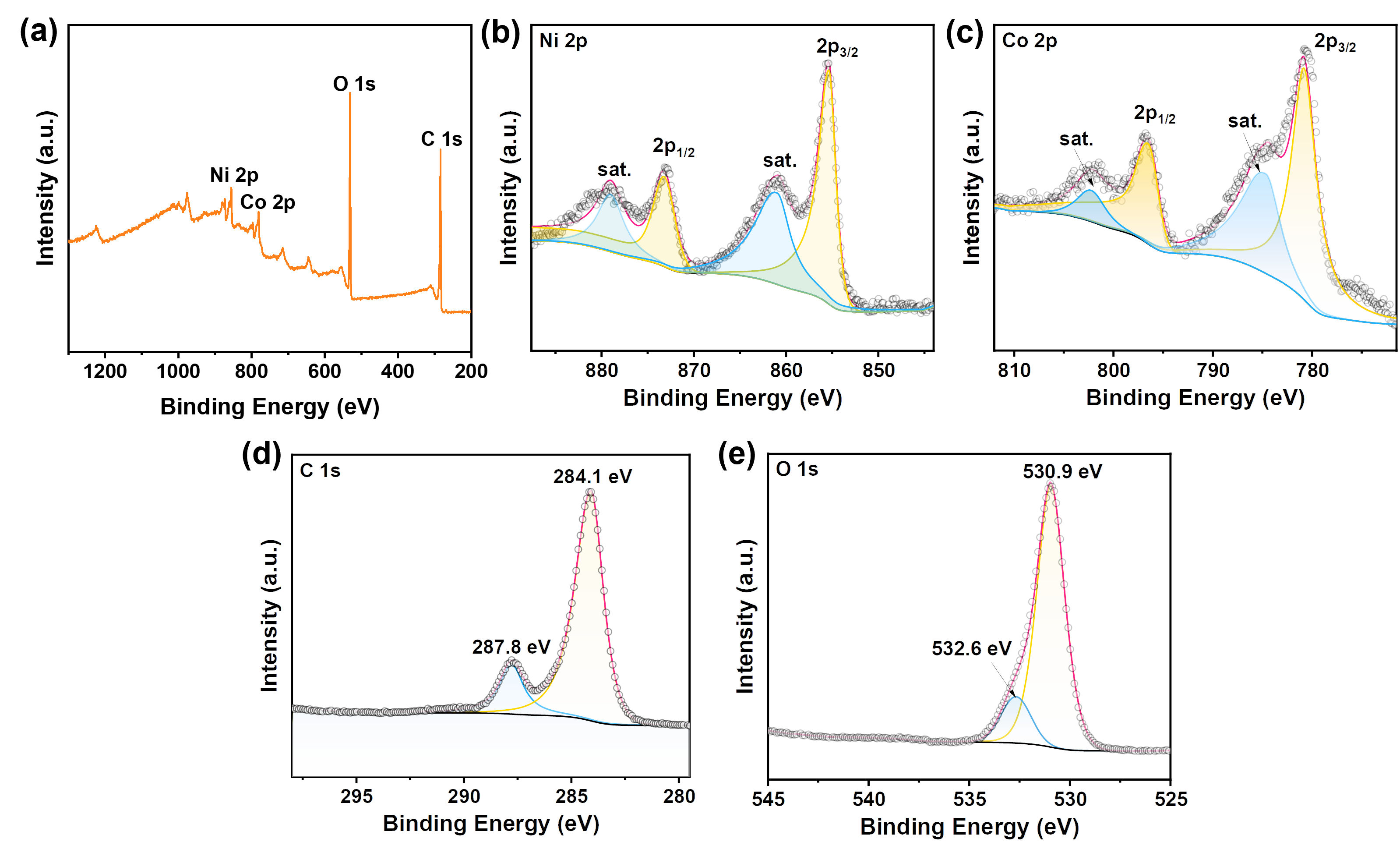


**Fig. S5.** (a) XPS survey spectrum and high-resolution (b) Ni 2p, (c) Co 2p, (d) C 1s, and (e) O 1s spectra of ML-MOF_(10:0)_.


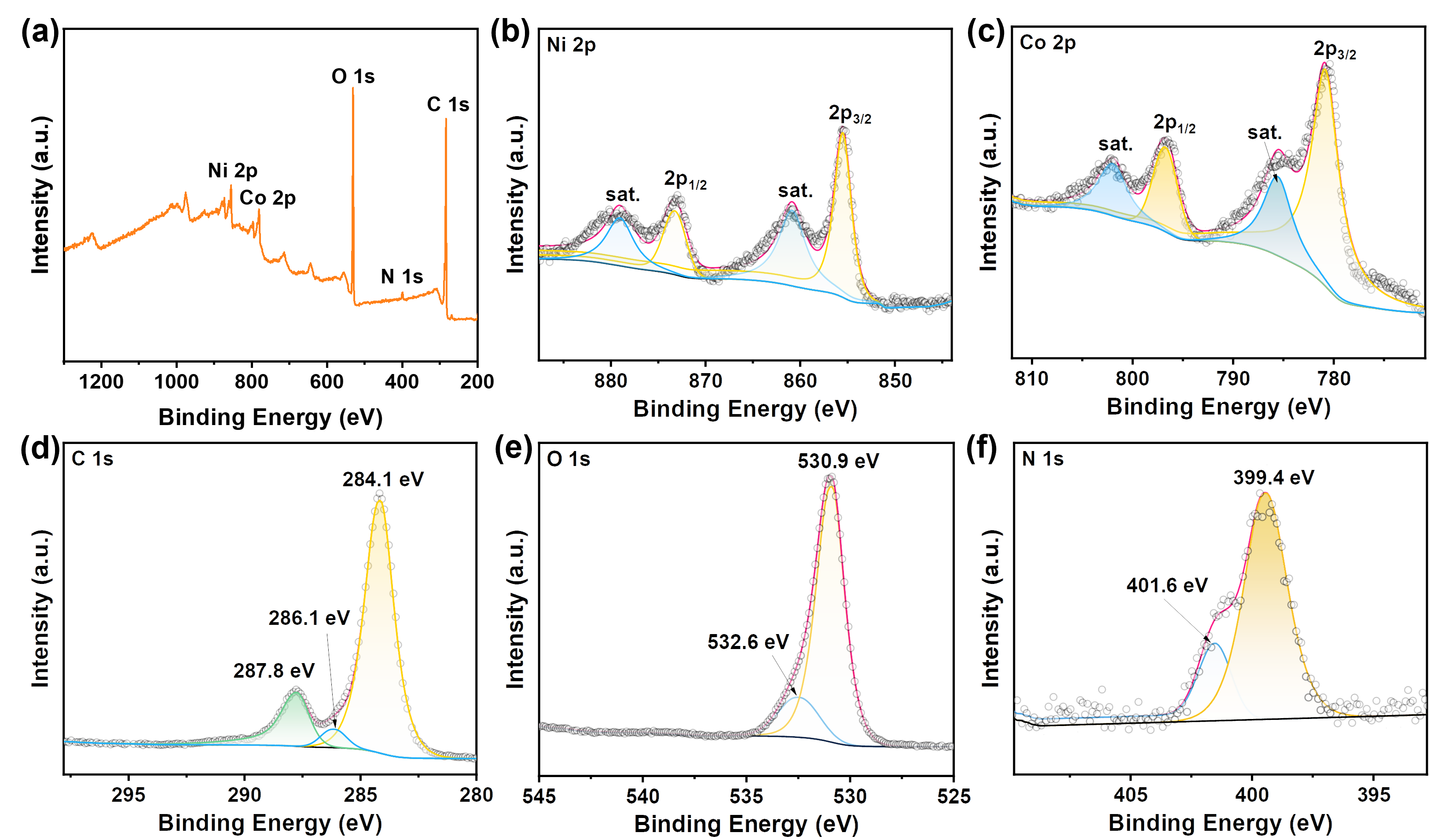


**Fig. S6.** (a) XPS survey spectrum and high-resolution (b) Ni 2p, (c) Co 2p, (d) C 1s, (e) O 1s, and (f) N 1s spectra of ML-MOF_(8:2)_.


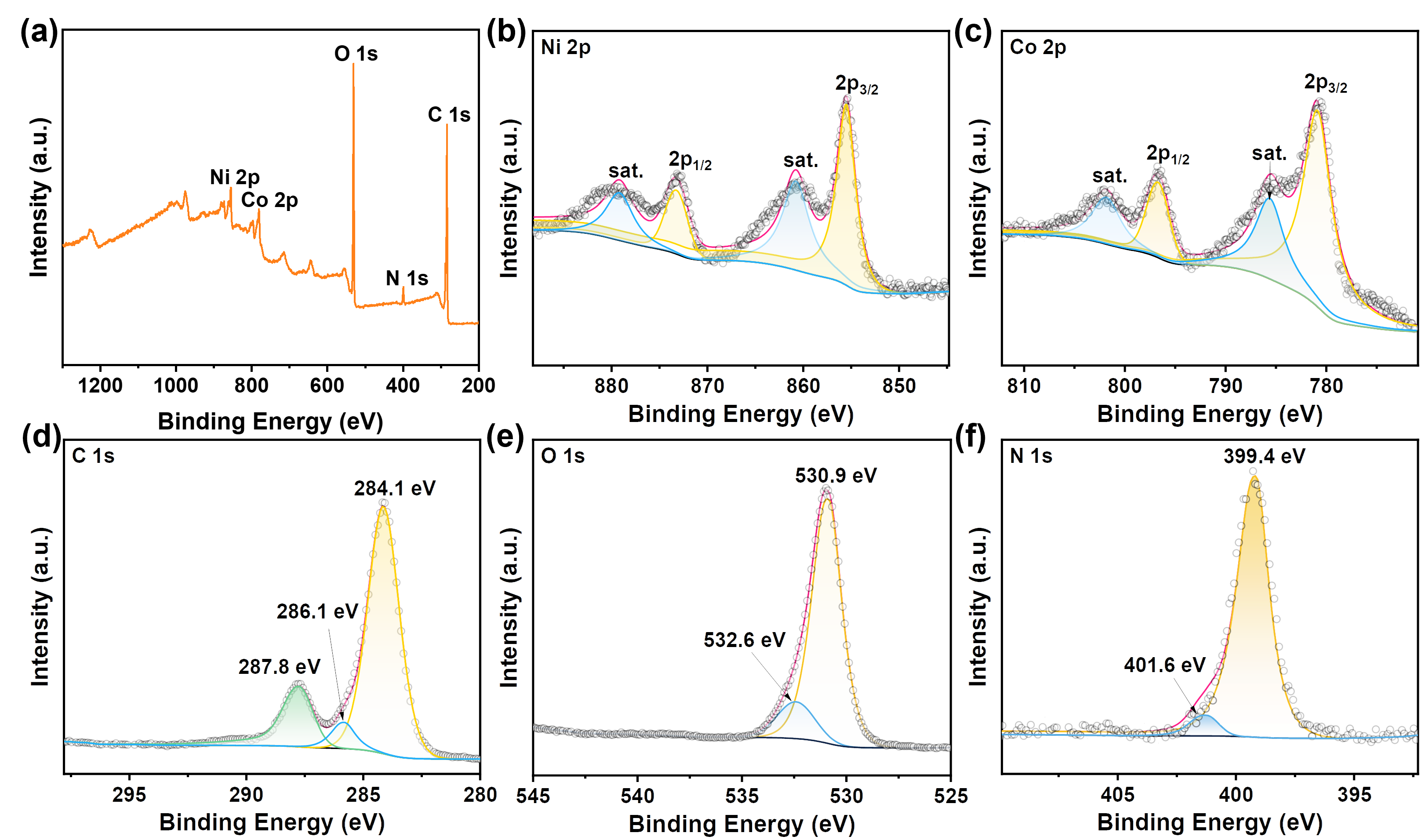


**Fig. S7.** (a) XPS survey spectrum and high-resolution (b) Ni 2p, (c) Co 2p, (d) C 1s, (e) O 1s, and (f) N 1s spectra of ML-MOF_(5:5)_.


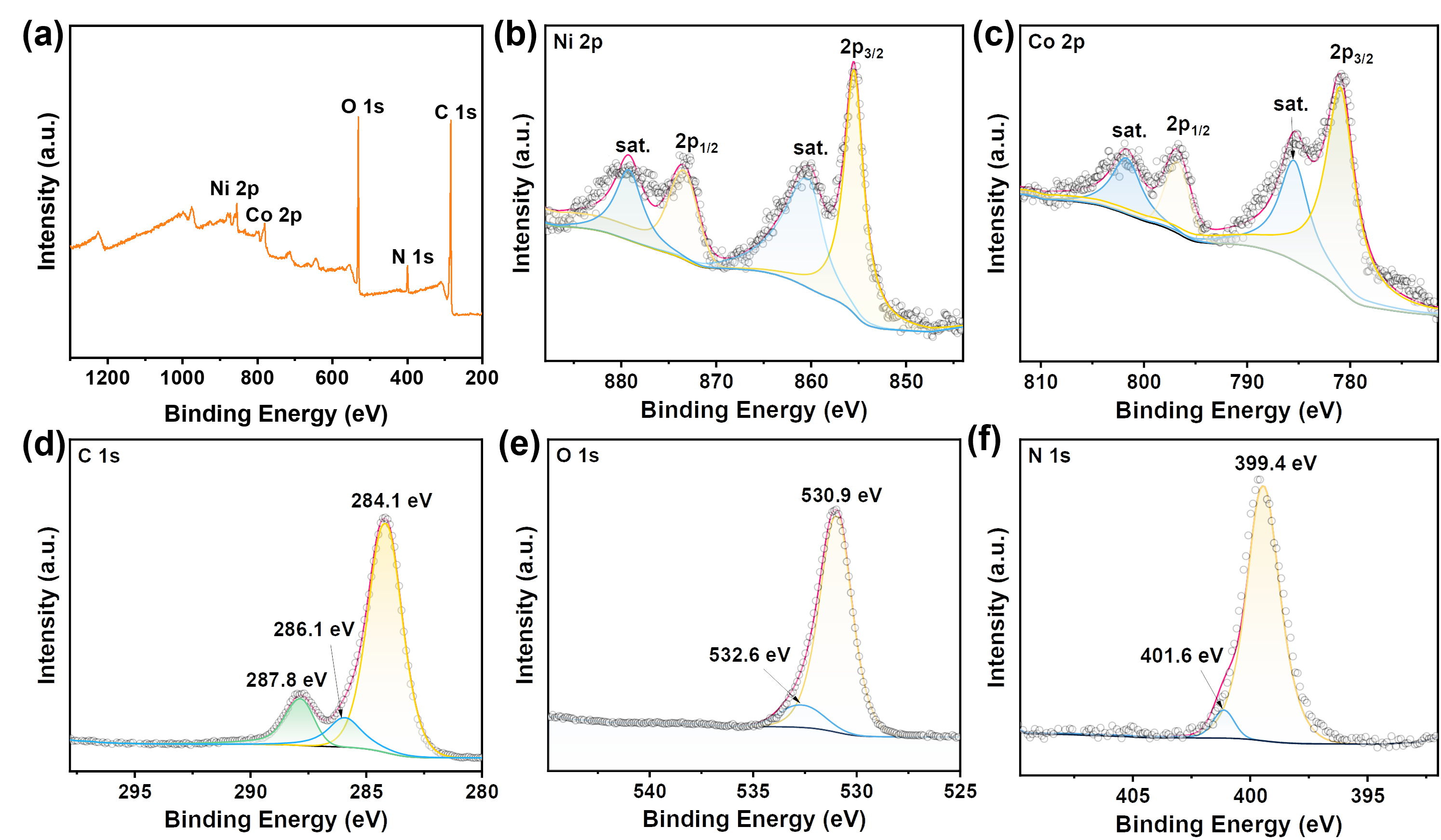


**Fig. S8.** (a) XPS survey spectrum and high-resolution (b) Ni 2p, (c) Co 2p, (d) C 1s, (e) O 1s, and (f) N 1s spectra of ML-MOF_(2:8)_.


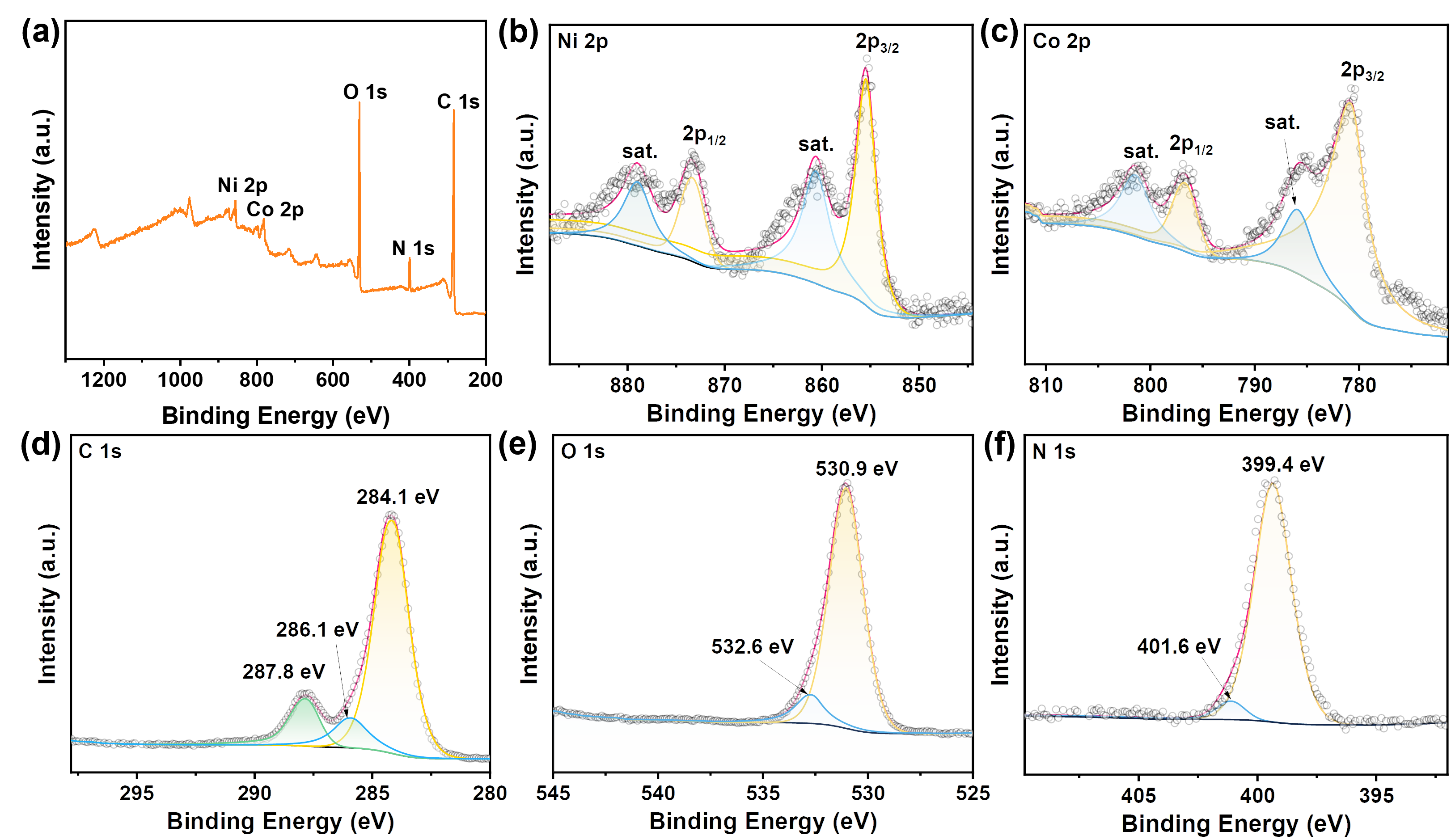


**Fig. S9.** (a) XPS survey spectrum and high-resolution (b) Ni 2p, (c) Co 2p, (d) C 1s, (e) O 1s, and (f) N 1s spectra of ML-MOF_(0:10)_.

The X-ray photoelectron spectroscopy (XPS) results for ML-MOF_(10:0)_, ML-MOF_(8:2)_, ML-MOF_(5:5)_, ML-MOF_(2:8)_, and ML-MOF_(0:10)_ are shown in Figs. S5-S9. XPS survey spectra indicate that ML-MOF_(10:0)_ contains Ni, Co, O, and C elements, while ML-MOF_(8:2)_, ML-MOF_(5:5)_, ML-MOF_(2:8)_, and ML-MOF_(0:10)_ contain Ni, Co, O, C, and N elements (Figs. S5a, S6a, S7a, S8a, and S9a). In the Ni 2p spectra (Figs. S5b, S6b, S7b, S8b, and S9b), the fitted peaks at approximately 855.6 eV and 873.3 eV correspond to Ni 2p 3/2 and Ni 2p 1/2, respectively, indicating the presence of Ni²⁺ species.^[10]^ XPS peaks at approximately 781.0 eV and 796.5 eV (Figs. S5c, S6c, S7c, S8c, and S9c) correspond to Co 2p 3/2 and Co 2p 1/2, confirming the presence of Co²⁺ species.^[11]^ The high-resolution C 1s spectrum of ML-MOF_(10:0)_ (Fig. S5d) can be deconvoluted into two peaks: C−C=C (284.1 eV) and O-C=O (287.8 eV).^[12-13]^ In contrast, the high-resolution C 1s spectra of ML-MOF_(8:2)_, ML-MOF_(5:5)_, ML-MOF_(2:8)_, and ML-MOF_(0:10)_ (Figs. S6d, S7d, S8d, S9d) display additional peaks at 286.1 eV (C-N),^[14]^ indicating the incorporation of NH_2_-BDC ligand in these samples. The O 1s XPS spectra (Figs. S5e, S6e, S7e, S8e, S9e) contain two characteristic peaks: M-O-M at 530.9 eV and -OH at 532.6 eV.^[15-16]^ Notably, the N 1s spectra of ML-MOF_(8:2)_, ML-MOF_(5:5)_, ML-MOF_(2:8)_, and ML-MOF_(0:10)_ exhibit characteristic -NH_2_ and -N-^+^ peaks (Figs. S6f, S7f, S8f, and S9f),^[17-18]^ further confirming the incorporation of NH_2_-BDC ligand in these samples.


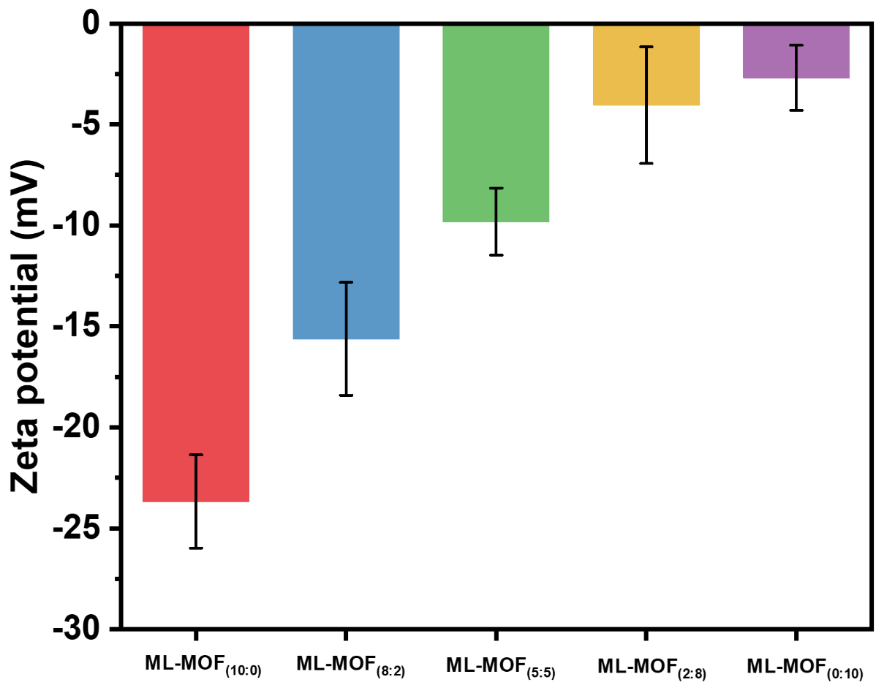


**Fig. S10.** Zeta potential measurements of ML-MOF nanoparticles with varying BDC/NH_2_-BDC ligand ratios (10:0, 8:2, 5:5, 2:8, 0:10).

Fig. S10 displays the zeta potential measurements of ML-MOF nanoparticles across different BDC/NH_2_-BDC ligand ratios. The data show a clear trend: as the NH_2_-BDC ligand ratio increases, the zeta potential shifts from -23.7 mV to -2.7 mV, with intermediate values of -15.7 mV, -9.8 mV, and -4.0 mV. This variation in zeta potential is attributed to the differing concentrations of carboxyl and amino functional groups in the ML-MOF nanoparticles.^[19]^ Specially, the increase in zeta potential is directly linked to the higher concentration of amino functional groups introduced by the NH_2_-BDC ligands.


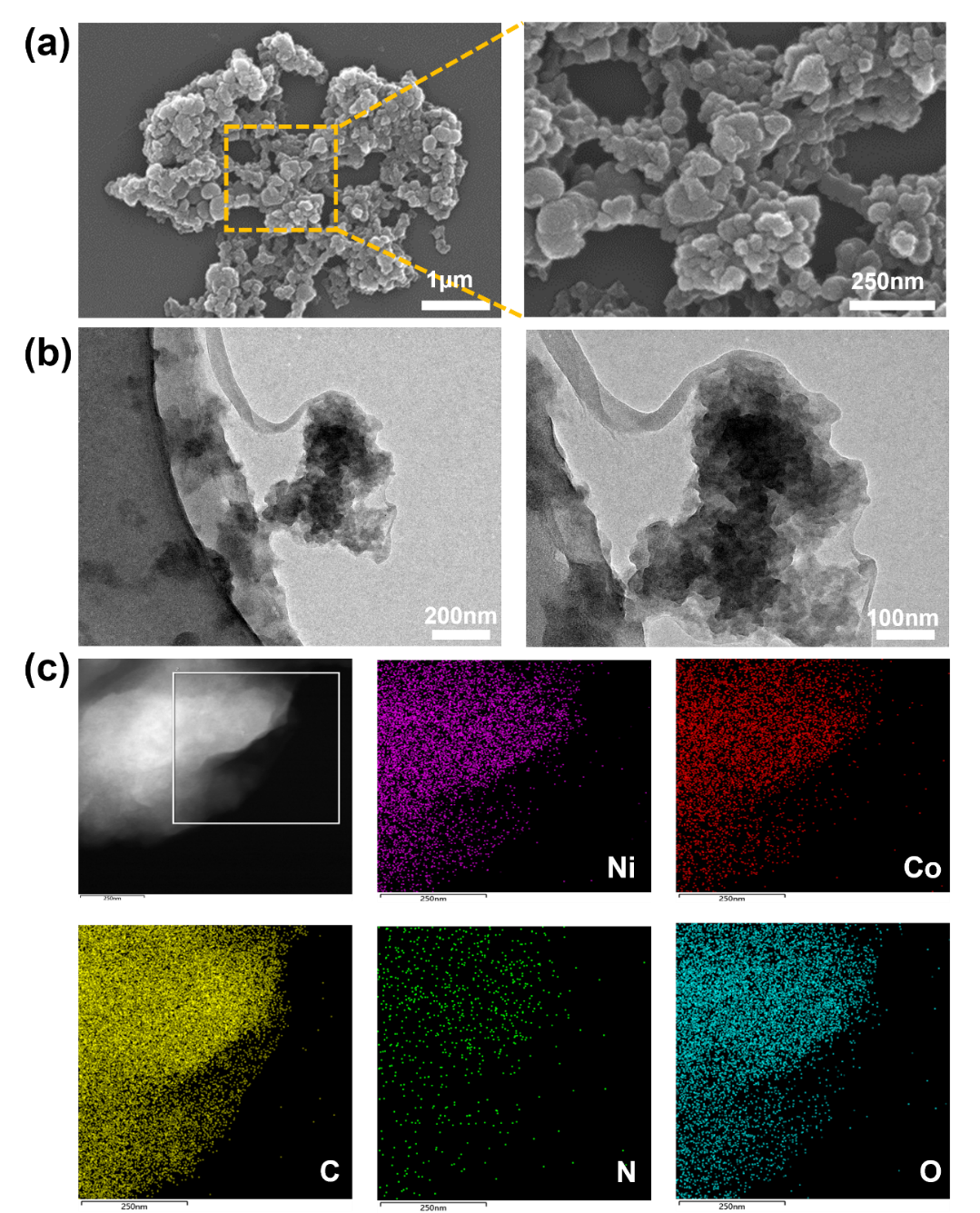


**Fig. S11.** Characterization of ML-MOF_(5:5)_ nanoparticles: (a) SEM images of ML-MOF_(5:5)_ nanoparticle; (b) EDS analysis showing the elements distribution in the ML-MOF_(5:5)_ nanoparticle; (c) TEM images of ML-MOF_(5:5)_ nanoparticle.

The microstructure and morphology of the ML-MOF_(5:5)_ nanoparticle were characterized using SEM and TEM, as shown in Fig. S11. SEM analysis revealed a granular morphology of the nanoparticle. TEM imaging indicated that the ML-MOF nanoparticles were approximately 500 nm in size. Elemental mapping analysis confirmed the uniform distribution of Ni, Co, N, C, and O within the nanoparticle. The Ni:Co ratio in the nanoparticle was approximately 1:1, consistent with the ratio of metal precursors used in the synthesis, thereby confirming the successful synthesis of the ML-MOF_(5:5)_ nanoparticle.


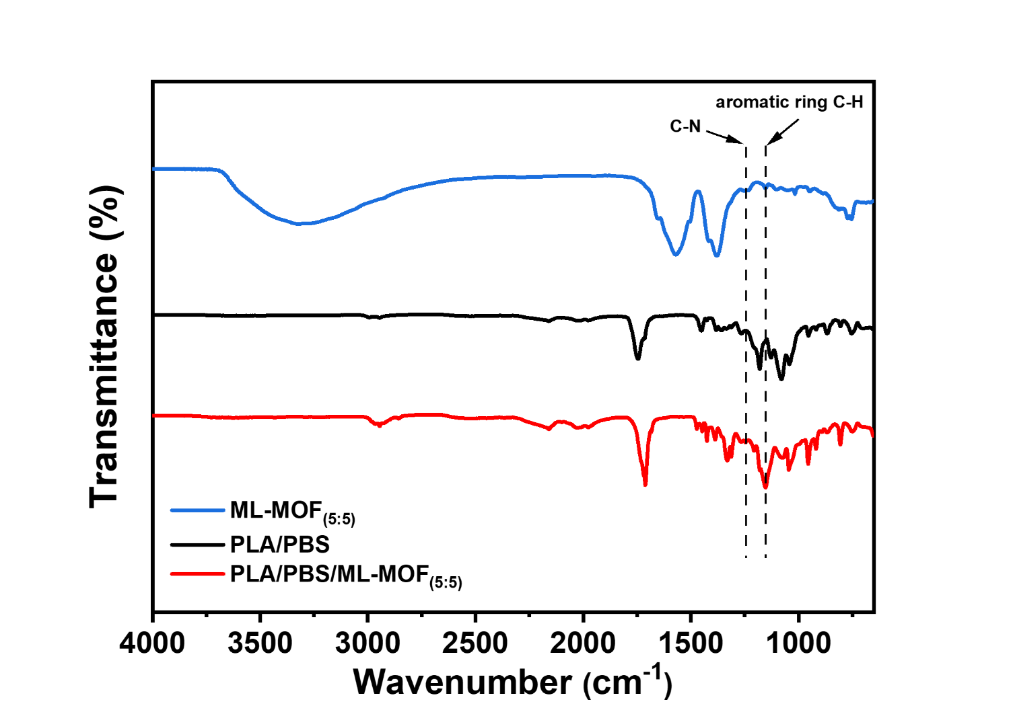


**Fig. S12.** FTIR spectra for ML-MOF_(5:5)_, PLA/PBS blend, and PLA/PBS/ML-MOF_(5:5)_ blend.

Fig. S12 shows the FTIR spectra of the ML-MOF_(5:5)_, PLA/PBS blend, and PLA/PBS/ML-MOF_(5:5)_ blend. Compared to the PLA/PBS blend, the PLA/PBS/ML-MOF_(5:5)_ blend exhibits a distinctive absorption peak for the C-N bond at 1245.8 cm⁻¹. Additionally, the peak at 1150.8 cm⁻¹ corresponds to the stretching vibrations of the C-H bond in the aromatic ring. These characteristic peaks confirm the successful incorporation of ML-MOF_(5:5)_ into the PLA/PBS blend.


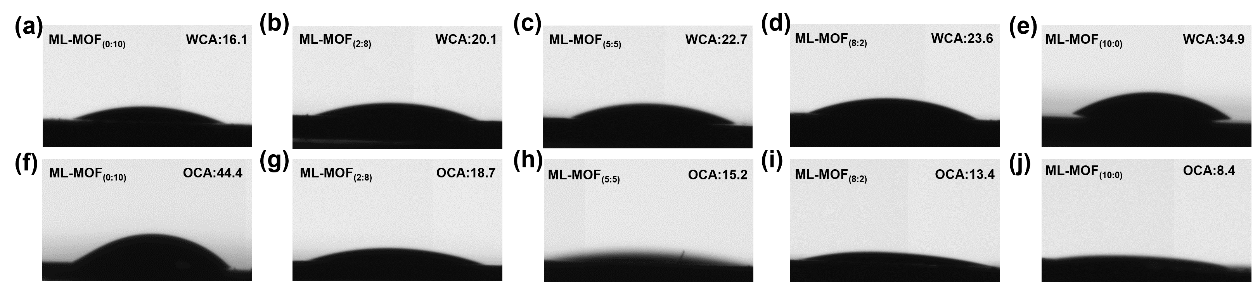


**Fig. S13.** Water and oil contact angles of ML-MOF nanoparticles with varying BDC/NH_2_-BDC ligand ratios.

Fig. S13 demonstrates that as the proportion of the hydrophilic ligand NH_2_-BDC decreases, the water contact angle (WCA) increases from 16.1° to 34.9°, and the oil contact angle (OCA) decreases from 44.4° to 8.4°. This trend indicates a gradual decline in hydrophilicity and a corresponding increase in oleophilicity of the ML-MOF nanoparticles.


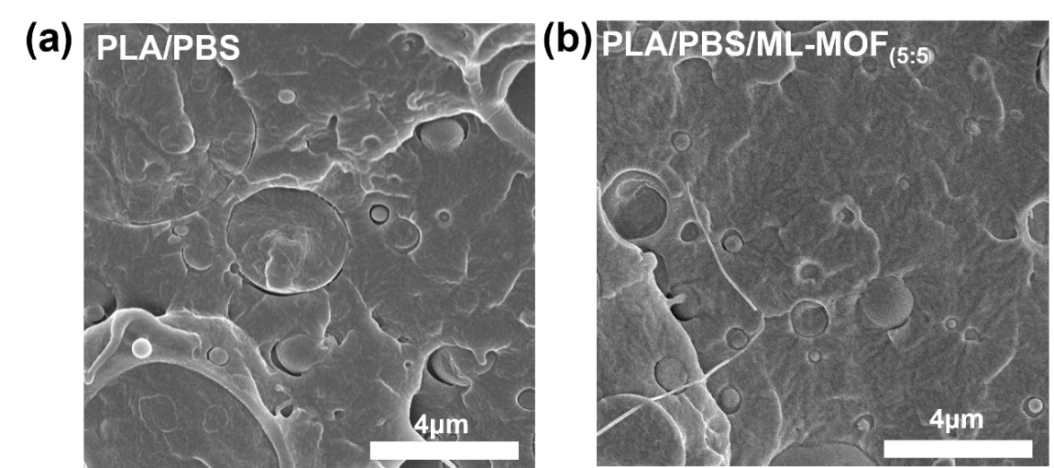


**Fig. S14.** SEM micrographs of PLA/PBS and PLA/PBS/ML-MOF_(5:5)_ blends at low magnification, illustrating the morphological differences between the two blends.

Fig. S14 demonstrates that incorporating ML-MOF_(5:5)_ nanoparticle into PLA/PBS blend significantly reduces the size and distribution of the dispersed phase, as observed at low magnifications. This indicates an improved compatibility and dispersion of the polymer phases due to the presence of ML-MOF_(5:5)_ nanoparticle.


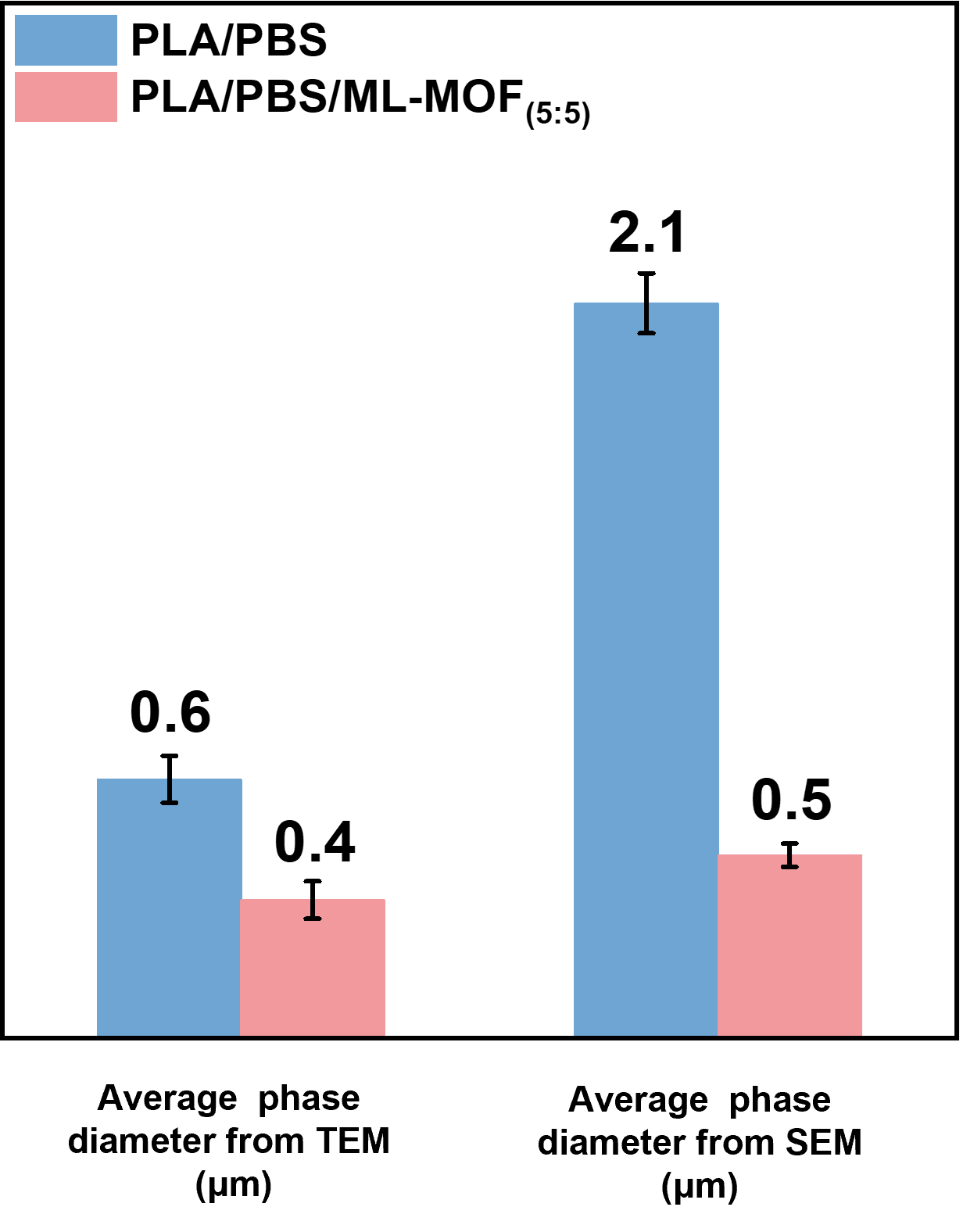


**Fig. S15.** Comparison of average PBS domain sizes in PLA/PBS and PLA/PBS/ML-MOF_(5:5)_ blends as observed from TEM and SEM images.

Fig. S15 reveals that the average size of the PBS dispersed phase in the PLA/PBS blend was 0.6 μm as observed in TEM images and 2.1 μm in SEM images. For the PLA/PBS/ML-MOF_(5:5)_ blend, the average sizes were signicantly reduced to 0.4 μm (TEM) and 0.5 μm (SEM), respectively. This reduction in domain size indicates improved dispersion and compatibility due to the incorporation of ML-MOF_(5:5)_ nanoparticle.


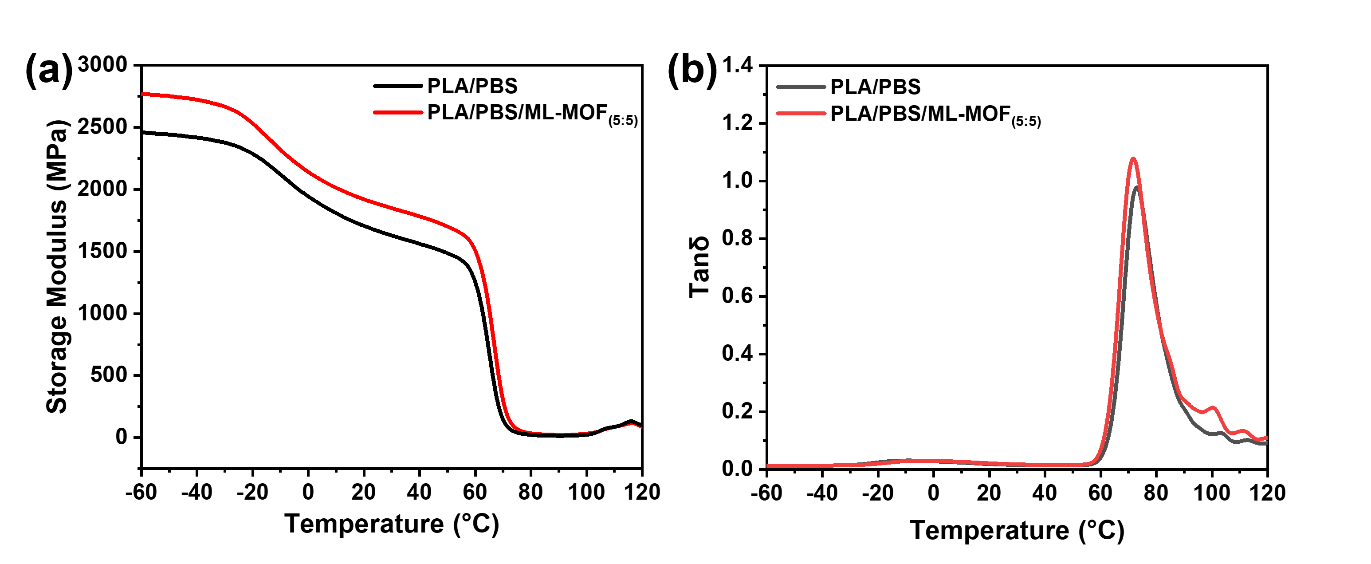
**Fig. S16.** DMA results of PLA/PBS and PLA/PBS/ML-MOF_(5:5)_ blends:

(a) Storage modulus and (b) Tan δ.

Fig. S16 illustrates the temperature-dependent storage modulus and tan δ for PLA/PBS and PLA/PBS/ML-MOF_(5:5)_ blends, as measured by DMA. Both blends show a decrease in storage modulus with increasing temperature, and two peaks in the tan δ curves indicating the glass transitions of PBS and PLA. Notably, the storage modulus of the PLA/PBS/ML-MOF_(5:5)_ blend exceeds that of PLA/PBS blend at room temperature, suggesting enhanced mechanical strength and compatibility. Additionally, the tan δ curve peaks reflect the glass transition temperatures (T_g_) of PBS and PLA, with T_g_ values at 84.6 °C for the PLA/PBS blend and 79.5 °C for the PLA/PBS/ML-MOF_(5:5)_ blend. These findings support the enhanced compatibility and mechanical performance of the PLA/PBS/ML-MOF_(5:5)_ blend, consistent with the previous SEM image observations.


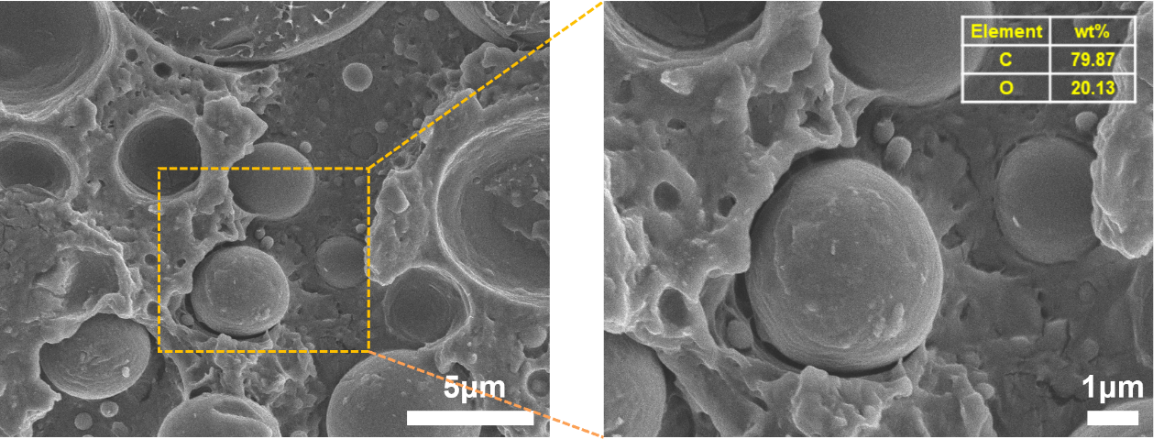


**Fig. S17.** Interface topography of the PLA/PBS blend.

Fig. S17 reveals the absence of nanoparticle distribution at the interface of the PBS and PLA phases in the PLA/PBS blend, resulting in visible gaps and indicating poor compatibility. Energy dispersive spectroscopy (EDS) analysis shows the elemental composition of C and O at the interface, confirming the absence of metal sources such as Ni and Co from the ML-MOF nanoparticle within the blend.


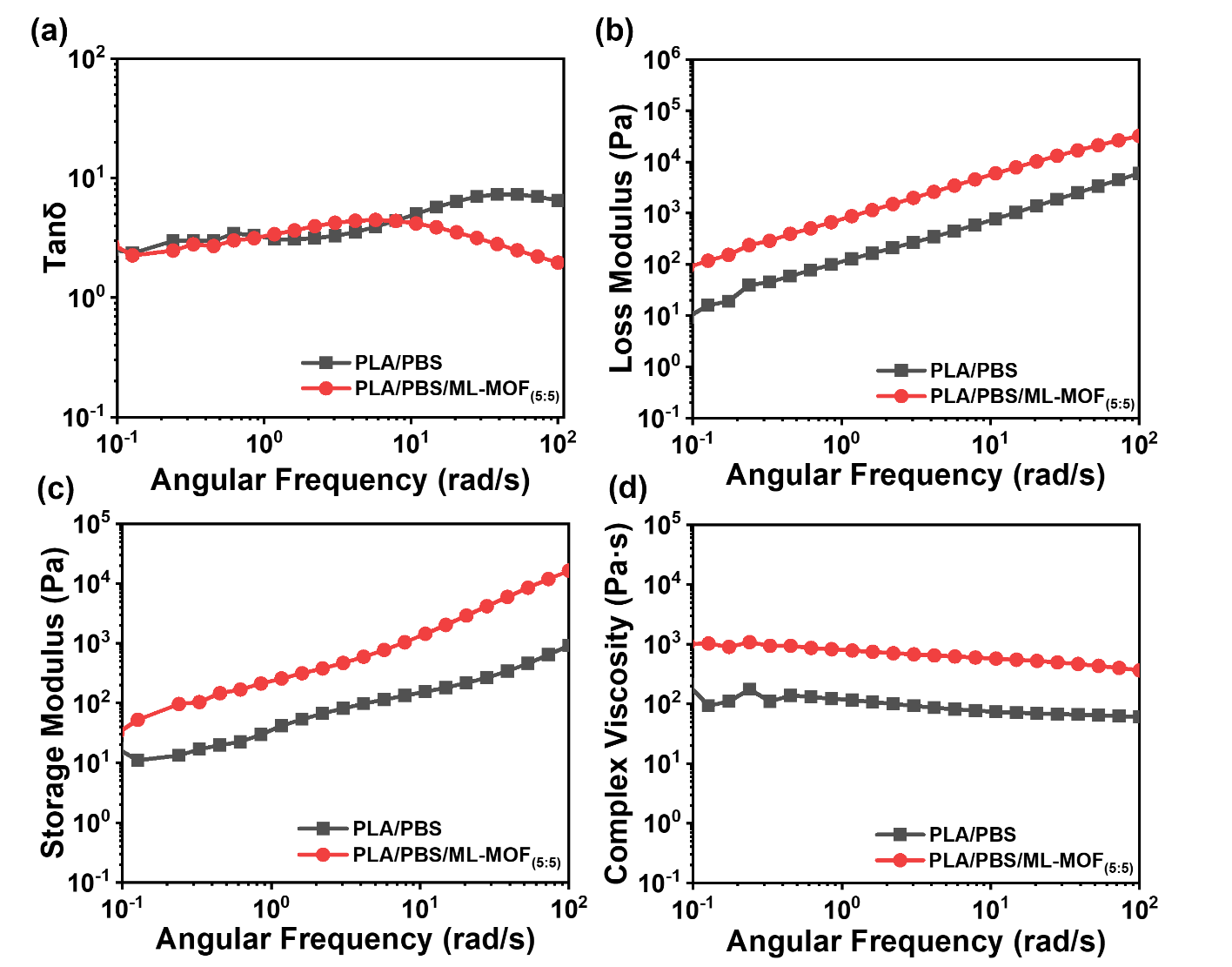


**Fig. S18.** Rheological properties of PLA/PBS and PLA/PBS/ML-MOF_(5:5)_ blends: (a) Tan δ, (b) Loss Modulus, (c) Storage Modulus, and (d) Complex Viscosity.

Fig. S18 shows frequency scan results from a rotational rheometer at 180 °C, indicating that PLA/PBS/ML-MOF_(5:5)_ blend exhibits higher complex viscosity than the PLA/PBS blend, with both materials demonstrating shear-thinning behavior. At 180 °C, the storage and loss moduli of PLA/PBS/ML-MOF_(5:5)_ blend are approximatly 0.5 and 1 order of magnitude greater than those of the PLA/PBS blend, respectively. The incorporation of ML-MOF_(5:5)_ nanoparticle leads to increased complex viscosity and moduli in PLA/PBS/ML-MOF_(5:5)_ due to intensified entanglement among molecular chains. In the low-frequency range, the tan δ values for both PLA/PBS and PLA/PBS/ML-MOF_(5:5)_ blends exceed 1, indicating viscous behavior. With increasing frequency, the tan δ of PLA/PBS/ML-MOF_(5:5)_ blend decreases, indicating a transition to solid-like properties suitable for processing with lower shear forces. Notably, the PLA/PBS blend exhibits two tan δ peaks, while the PLA/PBS/ML-MOF_(5:5)_ blend shows only one, reflecting enhanced microphase separation and compatibility in the blend.^[20]^


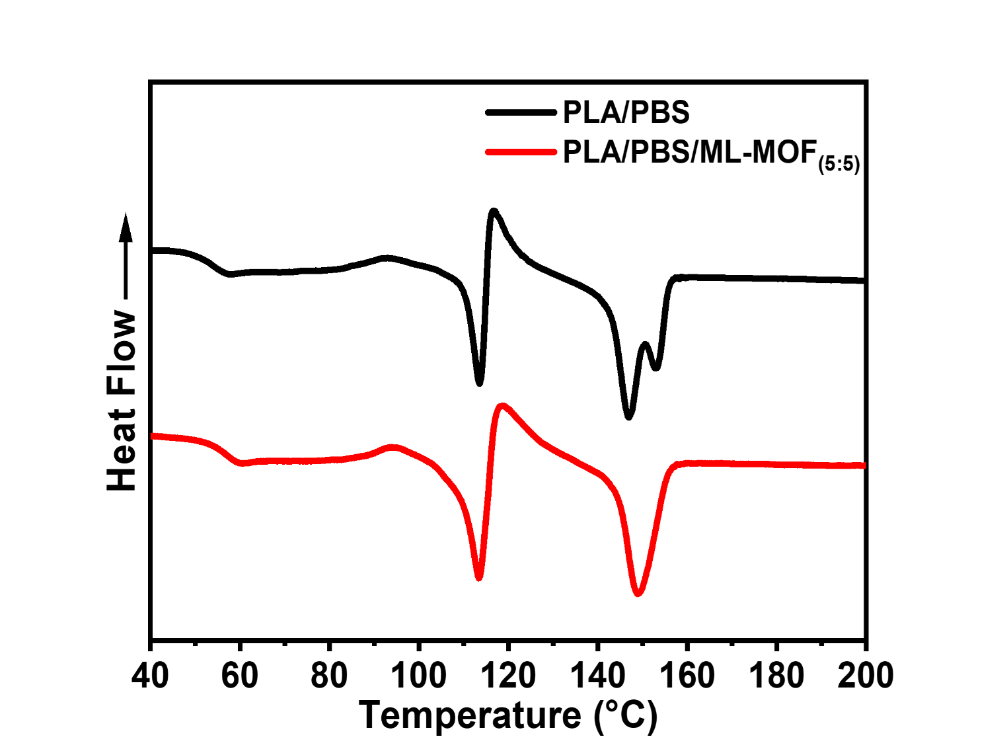


**Fig. S19.** DSC curves for PLA/PBS and PLA/PBS/ML-MOF_(5:5)_ blends.

DSC analysis was performed to examine the impact of ML-MOF_(5:5)_ nanoparticle on the crystallization behavior of PLA/PBS blend, with secondary heating curves shown in Fig. S19 and thermodynamic data in Table S3. The addition of ML-MOF_(5:5)_ nanoparticle slightly raised the crystallization and melting temperatures of the PLA phase in PLA/PBS/ML-MOF_(5:5)_ blend, also increasing its crystallinity. Conversely, the melting temperature of PBS phase marginally decreased, while its crystallinity increased. These effects are attributed to ML-MOF_(5:5)_ nanoparticle, which enhances non-homogeneous nucleation in both the PLA and PBS phases, thereby increasing the material's overall crystallinity. Additionally, without ML-MOF_(5:5)_ nanoparticle, a shoulder in melt peak of PBS phase suggested decomposition, which disappeared with the additoin of ML-MOF_(5:5)_ nanoparticle, improving the thermodynamic properties of PLA/PBS/ML-MOF_(5:5)_ blend. These findings align with heat resistance tests, affirming the positive effect of ML-MOF_(5:5)_ nanoparticle on thermal stability.


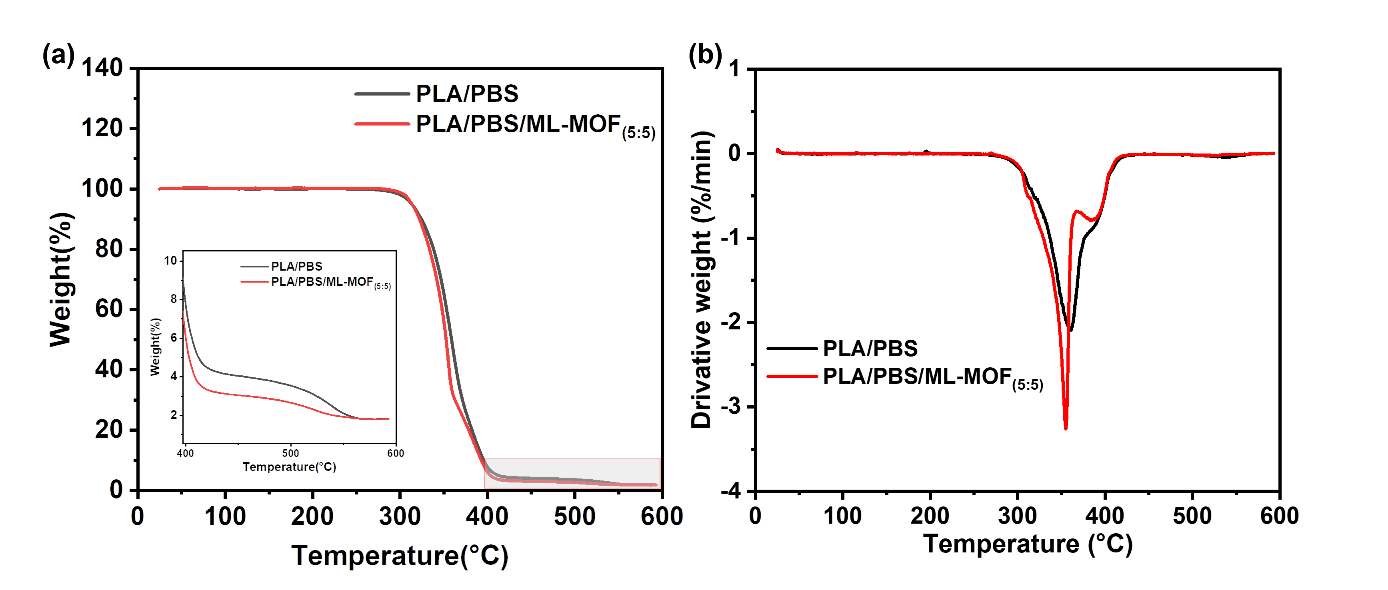


**Fig. S20.** TGA (a) and DTA (b) curves for PLA/PBS and PLA/PBS/ML-MOF_(5:5)_ blends.

The thermal stability of PLA/PBS and PLA/PBS/ML-MOF_(5:5)_ blends was assessed using TGA, as shown in Fig. S20 and summarized in Table S4. The addition of ML-MOF_(5:5)_ nanoparticle resulted in an increase in the initial thermal degradation temperature (T_d5%_) and a decrease in the maximum degradation temperature (T_max_). This effect is likely due to the formation of metal compounds during the thermal degradation of ML-MOF_(5:5)_ nanoparticle, which catalyzes PLA depolymerization, producing lactic acid and accelerating the thermal degradation of the PLA/PBS/ML-MOF_(5:5)_ blend.^[21]^ Consequently, incorporating ML-MOF_(5:5)_ nanoparticle significantly improves the blends' thermal stability, particularly from 20°C to 350°C.


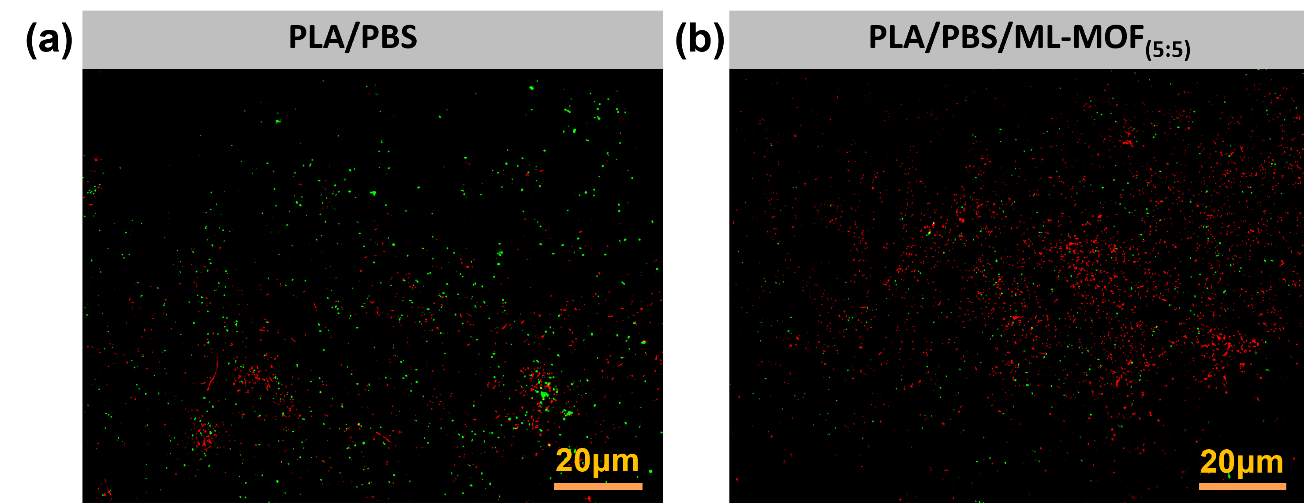


**Fig. S21.** Fluorescence microscopy images showing the live/dead *E. coli* density on the surfaces of PLA/PBS and PLA/PBS/ML-MOF(5:5) blends.

Fig. S21 demonstrates the evaluation of the antibacterial properties of PLA/PBS and PLA/PBS/ML-MOF_(5:5)_ blends against Escherichia coli (*E. coli*, a Gram-negative bacterium). The samples were immersed in a bacterial solution for 24 h, stained for fluorescence, and observed under an inverted fluorescence microscope to determine the density of live and dead bacteria. The results indicated that the PLA/PBS/ML-MOF_(5:5)_ blend had a higher count of dead bacteria and a lower count of live bacteria compared to the PLA/PBS blend, demonstrating good antibacterial performance.


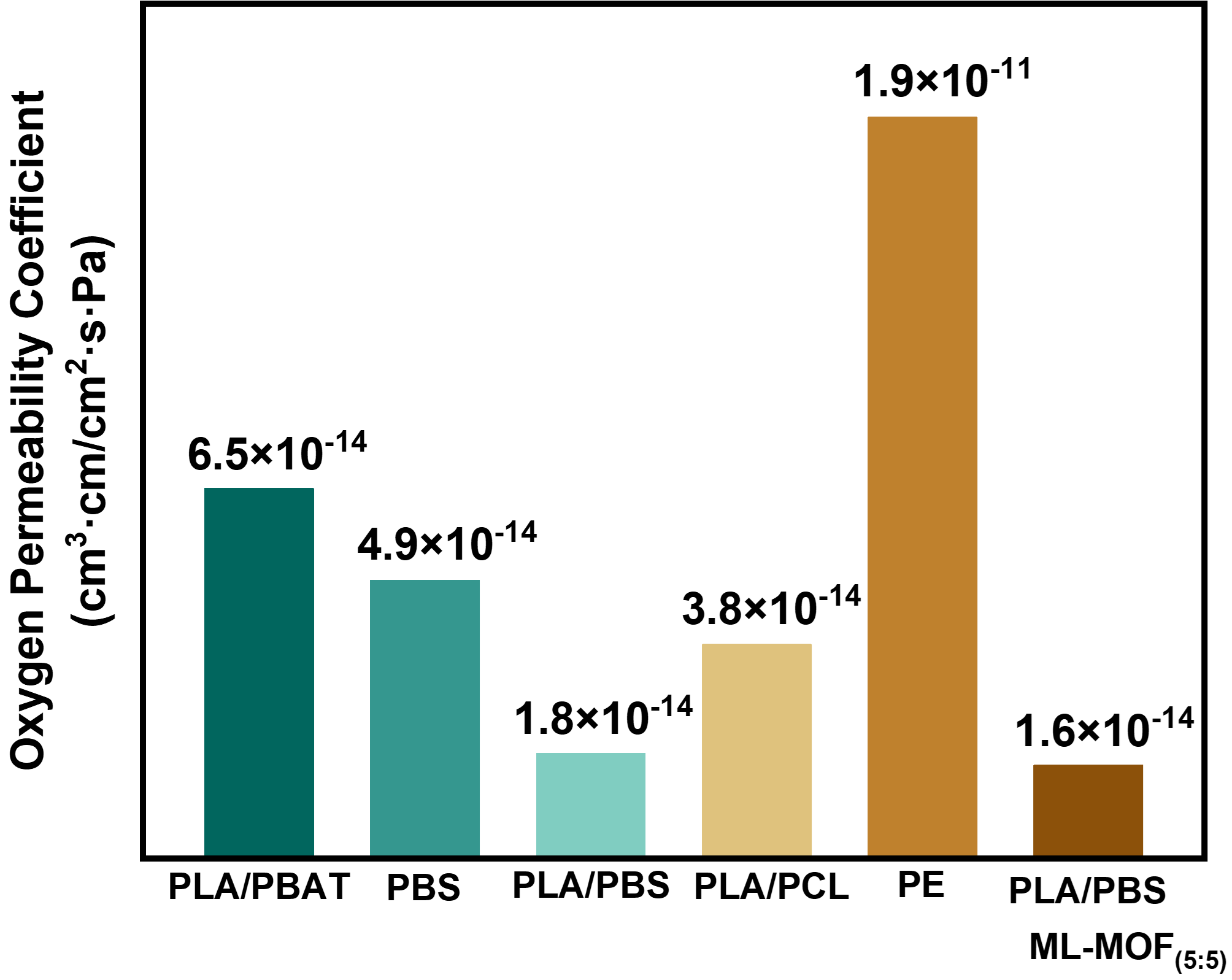


**Fig. S22.** Oxygen permeability coefficients of common food packaging materials (PLA/PBAT,^[22]^ PBS,^[23]^ PLA/PBS, PLA/PCL,^[24]^ PE^[25]^) compared to the PLA/PBS/ML-MOF_(5:5)_ blend.

Fig. S22 demonstrates that the PLA/PBS/ML-MOF_(5:5)_ blend has a lower oxygen permeability coefficient compared to common food packaging materials such as PLA/PBAT, PBS, PLA/PBS, PLA/PCL, and PE. This property indicates that the PLA/PBS/ML-MOF_(5:5)_ blend can act as an effective oxygen barrier, thereby preventing oxidative spoilage of food products.


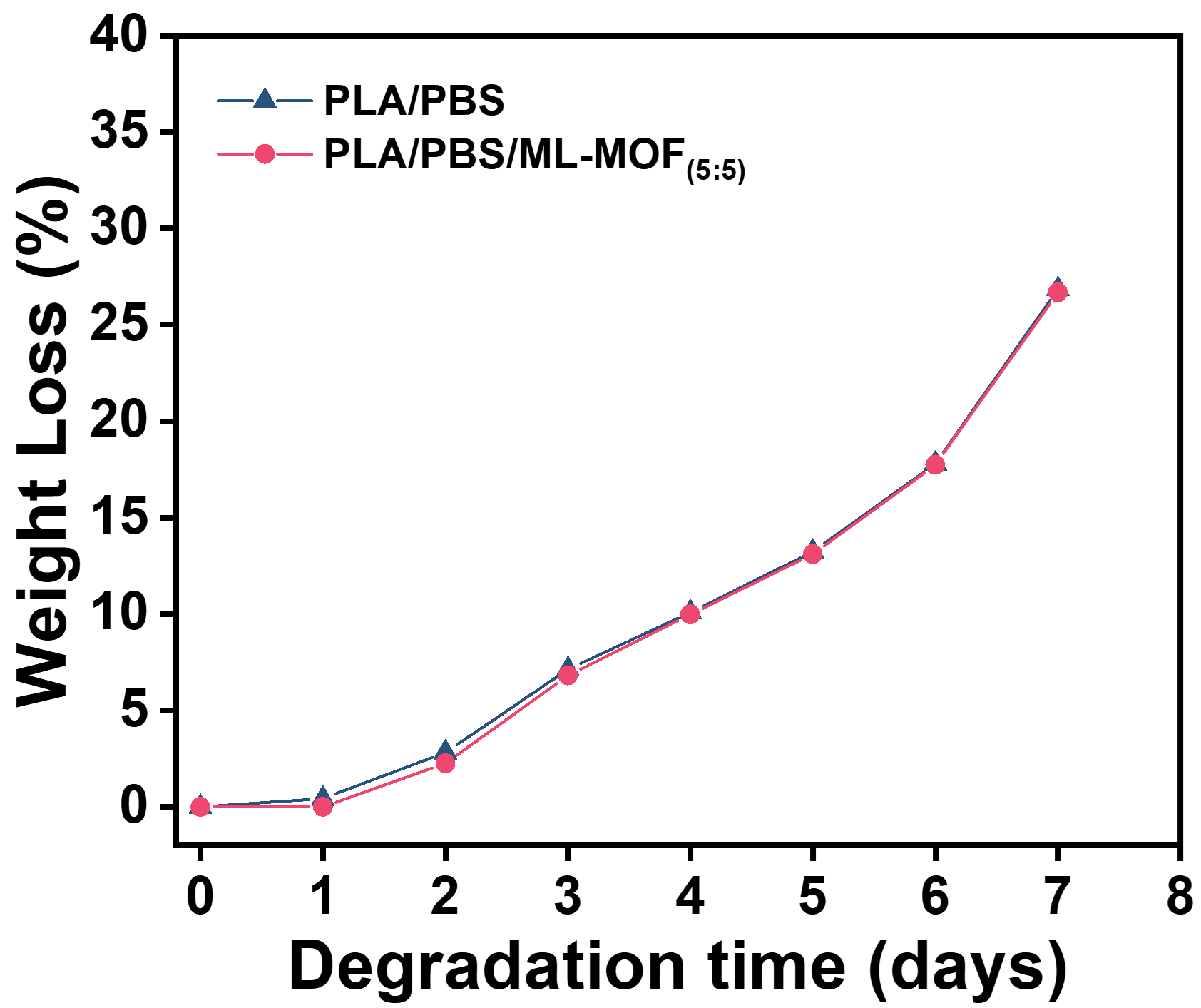


**Fig. S23.** Degradation curves of PLA/PBS and PLA/PBS/ML-MOF_(5:5)_ blends over time.

Fig. S23 illustrates the degradation profiles of PLA/PBS and PLA/PBS/ML-MOF_(5:5)_ blends. The weight loss curves indicate that the degradation rates of both PLA/PBS and PLA/PBS/ML-MOF_(5:5)_ blends steadily increased over time, reaching approximately 26% by the 7th day. Initially, from day 1 to day 3, PLA/PBS blend exhibited a slightly higher degradation rate compared to PLA/PBS/ML-MOF_(5:5)_ blend. However, from day 4, the degradation date of PLA/PBS/ML-MOF_(5:5)_ blend increased, and by the 7th day, the rates of both blends were nearly indentical. This suggests that the incorporation of ML-MOF_(5:5)_ nanoparticle does not significantly alter the degradability of the PLA/PBS blend. Consequently, PLA/PBS/ML-MOF_(5:5)_ blend maintains the biodegradability of the base polymer while potentially offering enhanced environmental friednliness, making it suitable for pratical applications.

**Table S1.** Force fields employed in MD simulations

|  | Melt State (T=1.5) | | | | Glass State (T=0.4) | | | |
| --- | --- | --- | --- | --- | --- | --- | --- | --- |
|  | pA | pB | oA | oB | pA | pB | oA | oB |
| pA | $\varepsilon_{\mathrm{WCA}}$=1 | $\varepsilon_{\mathrm{WCA}}$=2 | $\varepsilon_{\mathrm{LJ}}$=1 | $\varepsilon_{\mathrm{WCA}}$=2 | $\varepsilon_{\mathrm{LJ}}$=1 | $\varepsilon_{\mathrm{WCA}}$=2 | $\varepsilon_{\mathrm{LJ}}$=1 | $\varepsilon_{\mathrm{WCA}}$=2 |
| pB | $\varepsilon_{\mathrm{WCA}}$=2 | $\varepsilon_{\mathrm{WCA}}$=1 | $\varepsilon_{\mathrm{WCA}}$=2 | $\varepsilon_{\mathrm{LJ}}$=1 | $\varepsilon_{\mathrm{WCA}}$=2 | $\varepsilon_{\mathrm{LJ}}$=1 | $\varepsilon_{\mathrm{WCA}}$=2 | $\varepsilon_{\mathrm{LJ}}$=1 |
| oA | $\varepsilon_{\mathrm{LJ}}$=1 | $\varepsilon_{\mathrm{WCA}}$=2 | $\varepsilon_{\mathrm{WCA}}$=1 | $\varepsilon_{\mathrm{WCA}}$=1 | $\varepsilon_{\mathrm{LJ}}$=1 | $\varepsilon_{\mathrm{WCA}}$=2 | $\varepsilon_{\mathrm{WCA}}$=1 | $\varepsilon_{\mathrm{WCA}}$=1 |
| oB | $\varepsilon_{\mathrm{WCA}}$=2 | $\varepsilon_{\mathrm{LJ}}$=1 | $\varepsilon_{\mathrm{WCA}}$=1 | $\varepsilon_{\mathrm{WCA}}$=1 | $\varepsilon_{\mathrm{WCA}}$=2 | $\varepsilon_{\mathrm{LJ}}$=1 | $\varepsilon_{\mathrm{WCA}}$=1 | $\varepsilon_{\mathrm{WCA}}$=1 |

**Table S2.** Mechanical properties of PLA/PBS blends with varying ligand proportions of ML-MOF nanoparticles.

| **Sample** | **Tensile strength**  **(MPa)** | **Young's modulus**  **(MPa)** | **Elongation at break**  **(%)** |
| --- | --- | --- | --- |
| PLA/PBS | 48.1±2.0 | 944.5±5.8 | 9.2±1.0 |
| PLA/PBS/ML-MOF**_(0:10)_** | 50.3±1.3 | 1039.3±30.3 | 72.7±16.7 |
| PLA/PBS/ML-MOF**_(2:8)_** | 51.3±0.5 | 1047.2±27.2 | 123.6±30.0 |
| **PLA/PBS/ML-MOF_(5:5)_** | **53.3±0.9** | **1096.1±78.4** | **229.2±13.9** |
| PLA/PBS/ML-MOF**_(8:2)_** | 51.4±1.4 | 1060.2±28.1 | 157.7±27.9 |
| PLA/PBS/ML-MOF**_(10:0)_** | 52.0±1.4 | 1050.3±17.1 | 33.8±4.7 |

**Table S3.** Comparison of the relative increase in fracture elongation of PLA/PBS blends with different types and contents of compatibilizers.

| **Type** | **Compatibilizer** | **Content (wt%)** | **Original elongation at break**  **(%)** | **Improved elongation at break**  **(%)** | **The effect of improvement**  **(fold)** | **Ref.** |
| --- | --- | --- | --- | --- | --- | --- |
| / | **ML-MOF_(5:5)_** | **0.1** | **9.2** | **229.2** | **24.9** | **This work** |
| Copolymer | P(BS-co-LA) | 5 | 140.0 | 225.0 | 1.6 | 35 |
|  | PLLA-g-MA | 4 | 242.0 | 390.0 | 1.6 | 41 |
|  | PLA-g-MA | 1 | 39.5 | 4.9 | 0.1 | 42 |
|  | PBS-PLLA | 20 | 26.0 | 75.0 | 2.9 | 43 |
|  | rPBSL | 1 | 8.0 | 13.0 | 1.6 | 38 |
| Homopolymer | PEG | 6 | 6.0 | 7.8 | 1.3 | 44 |
|  | MFC-EPI | 2 | 91.9 | 273.6 | 2.9 | 45 |
|  | MFC-EPI | 3 | 91.9 | 227.3 | 2.5 | 45 |
| Inorganic filler | OMMT | 1 | 79.0 | 106.0 | 1.3 | 36 |
|  | Wood Flour | 30 | 105.0 | 5.0 | 0.1 | 37 |
|  | Talc | 20 | 5.6 | 4.4 | 0.8 | 39 |
| Low molecular compatibilizer | MDI | 5 | 24.7 | 285.0 | 11.5 | 40 |
|  | CBC | 2 | 242.0 | 286.0 | 1.2 | 41 |
|  | PBO | 2 | 242.0 | 398.0 | 1.6 | 41 |
|  | BPO | 1 | 275.0 | 390.0 | 1.4 | 34 |
|  | TDI | 1 | 39.5 | 2.3 | 0.1 | 42 |

**Table S4.** Comparison of ductility, strength, thermal stability, oxygen barrier and antibacterial properties of common polymer packaging materials with those of PLA/PBS/ML-MOF_(5:5)_ blend.

| **Sample** | **Ductility**  **(%)** | **Tensile**  **Strength**  **(MPa)** | **Thermal stability**  **(°C)** | **Antibacterial property**  **(%)** | **Antioxidant activity**  **(%)** | **Oxygen Permeability Coefficient**  **(10^-14^·cm^3^·cm/cm^2^·s·Pa)** | **Ref.** |
| --- | --- | --- | --- | --- | --- | --- | --- |
| PE | 180.0 | 35.0 | 106.0 | 0 | 0 | 1900 | 28 |
| PVA | —— | —— | —— | 88.2 | —— | —— | 29 |
| PBS | 80.0 | 20.0 | 91.0 | 30.0 | —— | 4.9 | 32 |
| PLA | 2.6 | 36.0 | —— | —— | 0.84 | —— | 26 |
| PBAT | —— | —— | —— | 18.0 | 0 | —— | 31 |
| PLA/PBAT | 55.0 | 42.0 | 145.0 | —— | —— | 6.5 | 30 |
| PLA/PCL | 150.0 | 17.0 | 162.0 | —— | —— | 3.8 | 33 |
| PLA/PBS | 9.1 | 48.1 | 147.0 | 61.2 | 5.5 | 1.8 | 27 |
| **PLA/PBS/ML-MOF_(5:5)_** | **229.2** | **53.3** | **148.9** | **84.6** | **12.5** | **1.6** | **This Work** |

**Table S5.** Effect of ML-MOF_(5:5)_ nanoparticle on the crystallization behavior of PLA/PBS blend.

| **Sample** | **T_g_(PLA)**  **(°C)** | **T_cc_(PLA)**  **(°C)** | **T_m_(PBS)**  **(°C)** | **T_m_(PLA)**  **(°C)** | **ΔH_cc(PLA)_**  **(J/g)** | **ΔH_m_(PBS)**  **(J/g)** | **ΔH_m_(PLA)**  **(J/g)** | ***X*_C_(PLA)**  **(%)** | ***X*_C_(PBS)**  **(%)** |
| --- | --- | --- | --- | --- | --- | --- | --- | --- | --- |
| PLA/PBS | 55.6 | 93.0 | 113.6 | 146.9 | 4.5 | 6.7 | 17.2 | 19.4 | 11.2 |
| **PLA/PBS/ML-MOF_(5:5)_** | **57.5** | **94.0** | **113.4** | **148.9** | **3.3** | **10.9** | **18.1** | **22.6** | **18.2** |

**Table S6.** Effect of ML-MOF nanoparticle on the thermal stability of PLA/PBS blend.

| **Sample** | **T_d5%_**  **(℃)** | **T_d50%_**  **(℃)** | **DTG peak**  **(℃)** |
| --- | --- | --- | --- |
| PLA/PBS | 310.2 | 357.3 | 357.9 |
| **PLA/PBS/ML-MOF_(5:5)_** | **314.4** | **352.6** | **354.6** |

***Supplementary References:***

1. A. Mesbah, P. Rabu, R. Sibille, S. Lebègue, T. Mazet, B. Malaman, M. François, *Inorg. Chem.*, **2014**, 53, 872-881.
2. Shang. L, Wen. S, Liu. D, Chen. J, Mu. M, Duan. X, Xu. M, Yang. J, Wu. Y, Zhao. B, *Sensors and Actuators B: Chemical*, **2024**, 417, 136075.
3. Gamage. N.-D. H, McDonald. K. A, Matzger. A. J, *Angewandte Chemie International Edition*, **2016**, 55 (39), 12099-12103.
4. Cong. T, Huang. H, Zhang. H, Li. C, Zhao. Y, Fan. Z, Pan. L, *Colloids and Surfaces A: Physicochemical and Engineering Aspects*, **2022**, 651, 129777.
5. Sk. S, Madhu. R, Gavali. D. S, Bhasin. V, Thapa. R, Jha. S. N, Bhattacharyya. D, Kundu. S, Pal. U, *Journal of Materials Chemistry A*, **2023**, 11 (19), 10309-10318.
6. Gentile. F. S, Pannico. M, Causà. M, Mensitieri. G, Di Palma. G, Scherillo. G, Musto. P, J*ournal of Materials Chemistry A*, **2020**, 8 (21), 10796-10812.
7. Dang. T, Zhang. G, Li. Q, Cao. Z, Zhang. G, Duan. H, *Journal of Colloid and Interface Science*, **2020**, 577, 368-378.
8. Zeng. D, Zhu. G, Xia. C, *Fuel Processing Technology*, **2022**, 235, 107374.
9. Chu. R, Guo. X, Zhang. Y, Zhao. X, Xiong. X, Zhang. X, Zhang. F, Zhang. K, Wang. Y, *Materials Today Communications*, **2024**, 38, 107676.
10. Dao. X, Nie. M, Sun. H, Dong. W, Xue. Z, Li. Q, Liao. J, Wang. X, Zhao. X, Yang. D, *International Journal of Hydrogen Energy*, **2022**, 47 (38), 16741-16749.
11. Wei. W, Luo. S, Zhao. Y, Li. X, Liang. B, Fang. J, Luo, M. *New Journal of Chemistry*, **2021**, 45 (25), 11174-11182.
12. Wang. Q, Han. C, Tang. G, Liu. L, Li. T, Han. Y, Journal of Alloys and Compounds 2023, 931, 167510.
13. Chen. Y, Zhai. B, Liang. Y, *Diamond and Related Materials*, **2019**, 98, 107508.
14. Tang. B, Wang. S, Li. R, Gou. X, Long. J, *Journal of Power Sources*, **2019**, 425, 76-86.
15. Li. R, Che. R, Liu. Q, Su. S, Li. Z, Zhang. H, Liu. J, Liu. L, Wang, J. *Journal of Hazardous Materials*, **2017**, 338, 167-176.
16. He. Y, Lin. Y, Li. Y, Wang. C, *International Journal of Hydrogen Energy*, **2024**, 51, 1606-1615.
17. Gao. Z, Zhang. X, Xu. P, Sun. J, *Inorganic Chemistry Frontiers*, **2020**, 7 (10), 1995-2005.
18. Cao. M, Yang. F, Zhang. Q, Zhang. J, Zhang. L, Li. L, Wang. X, Dai. W.-L, *Journal of Materials Science & Technology*, **2021**, 76, 189-199.
19. He H, Liang F, *Chem. Mater.*, **2022**, 34(8): 3806-3818.
20. Wang D, Lu X, Qu J, *Polym. Composites*, **2018**, 39(9): 3057-3065.
21. X. Dai, Y. Cao, X. Shi, X. Wang, *RSC Adv.*, **2016**, 6(75): 71461-71471.
22. N. Jaouadi, R. Al-Itry, A. Maazouz, K. Lamnawar, *Polymers*, **2023**, 15(9): 2068.
23. A. R. de Matos Costa, A. Crocitti, L. Hecker de Carvalho, S. C. Carroccio, P. Cerruti, G. Santagata, *Polymers*, **2020**, 12: 2317.
24. Abd Al-Ghani M M, Azzam R A, Madkour T M, *Polymers*, **2021**, 13(20): 3527.
25. B. Jiang, Y. Tang, K. Zhou, L. Lu, X. Qiu, L. Pan, *J. Appl. Polym. Sci.*, **2023**, 140(1): e53243.
26. Byun Y, Kim Y T, Whiteside S, *J. Food Eng.*, **2010**, 100(2): 239-244.
27. S. Boonyod, W. Pivsa-Art, P. Nanthananon, Y. K. Kwon, S. Pivsa-Art, *J. Polym. Environ.*, **2023**, 31(7): 3070-3080.
28. Zhang Z, Han X, *Mater. Sci. Eng. C*, **2019**, 105: 110088.
29. W. Ma, L. Li, J. Li, X. Ren, Z.-G. Gu, T.-S. Huang, *Adv. Polym. Technol.*, **2018**, 37(5): 1390-1400.
30. X. Wang, L. Cui, S. Fan, X. Li, Y. Liu, *Polymers*, **2021**, 13(4): 507.
31. L. Xiao, Z. Yao, Y. He, Z. Han, X. Zhang, C. Li, P. Xu, W. Yang, P. Ma, *Ind. Crop. Prod.*, **2022**, 187: 115515.
32. Wattanawong N, Aht-Ong D, *Poly. Deg. Stab.*, **2021**, 183: 109459.
33. M. E. Diken, B. Koçer Kizilduman, S. Doğan, M. Doğan, *J. Appl. Polym. Sci.*, **2022**, 139(25): e52423.
34. Hu, X.; Su, T.; Li, P.; Wang, Z., *Polym. Bull.* **2018**, 75 (2), 533-546.
35. Zhang, W.; Xu, Y.; Wang, P.; Hong, J.; Liu, J.; Ji, J.; Chu, P. K., *J. Polym. Environ.* **2018**, 26 (7), 3060-3068.
36. Tan, L.-c.; He, Y.; Qu, J. p. J. P., *Polymer* **2019**, 180, 121656.
37. Nazrin, A.; Sapuan, S. M.; Zuhri, M. Y. M.; Ilyas, R. A.; Syafiq, R.; Sherwani, S. F. K., *Front. Chem.* **2020**, 8, 213.
38. Supthanyakul, R.; Kaabbuathong, N.; Chirachanchai, S., *Polymer* **2016**, 105, 1-9.
39. Pivsa-Art, W.; Pivsa-Art, S., *J. Polym. Environ.* **2019**, 27 (8), 1821-1827.
40. Li, L.; Song, G.; Tang, G., *Polym. Plast. Technol. Eng.* **2013**, 52 (12), 1183-1187.
41. Persenaire, O.; Quintana, R.; Lemmouchi, Y.; Sampson, J.; Martin, S.; Bonnaud, L.; Dubois, P., *Polym. Int.* **2014**, 63, 1724-1731.
42. Phetwarotai, W.; Maneechot, H.; Kalkornsurapranee, E.; Phusunti, N., *Polym. Adv. Technol.* **2018**, 29 (7), 2121-2133.
43. Zhang, B.; Sun, B.; Bian, X.; Li, G.; Chen, X., *Ind. Eng. Chem. Res.* **2017**, 56 (1), 52-62.
44. Pivsa-Art, W.; Fujii, K.; Nomura, K.; Aso, Y.; Ohara, H.; Yamane, H., *J. Appl. Polym. Sci.* **2016**, 133 (8), 43044.
45. He, L.; Song, F.; Li, D.-F.; Zhao, X.; Wang, X.-L.; Wang, Y.-Z., *ACS Sustain. Chem. Eng.* **2020**, 8 (3), 1573-1582.
